# Leo1 is essential for the dynamic regulation of heterochromatin and gene expression during cellular quiescence

**DOI:** 10.1186/s13072-019-0292-7

**Published:** 2019-07-17

**Authors:** Eriko Oya, Mickaël Durand-Dubief, Adiel Cohen, Vladimir Maksimov, Catherine Schurra, Jun-ichi Nakayama, Ronit Weisman, Benoit Arcangioli, Karl Ekwall

**Affiliations:** 1 Department of Biosciences and Nutrition, Karolinska Institutet, NEO Building, 141 83 Huddinge, Sweden; 2 Department of Natural and Life Sciences, The Open University of Israel, Ra’anana, Israel; 3 Unite Dynamique du Génome, Département Génomes et Génétique, Pasteur Institute, Paris, France; 4 Division of Chromatin Regulation, National Institute for Basic Biology, Okazaki, Japan

**Keywords:** Paf1C, Cellular quiescence, Heterochromatin, Gene expression, Fission yeast

## Abstract

**Background:**

Cellular quiescence is a reversible differentiation state during which cells modify their gene expression program to inhibit metabolic functions and adapt to a new cellular environment. The epigenetic changes accompanying these alterations are not well understood. We used fission yeast cells as a model to study the regulation of quiescence. When these cells are starved for nitrogen, the cell cycle is arrested in G1, and the cells enter quiescence (G0). A gene regulatory program is initiated, including downregulation of thousands of genes—for example, those related to cell proliferation—and upregulation of specific genes—for example, autophagy genes—needed to adapt to the physiological challenge. These changes in gene expression are accompanied by a marked alteration of nuclear organization and chromatin structure.

**Results:**

Here, we investigated the role of Leo1, a subunit of the conserved RNA polymerase-associated factor 1 (Paf1) complex, in the quiescence process using fission yeast as the model organism. Heterochromatic regions became very dynamic in fission yeast in G0 during nitrogen starvation. The reduction of heterochromatin in early G0 was correlated with reduced target of rapamycin complex 2 (TORC2) signaling. We demonstrated that cells lacking Leo1 show reduced survival in G0. In these cells, heterochromatic regions, including subtelomeres, were stabilized, and the expression of many genes, including membrane transport genes, was abrogated. TOR inhibition mimics the effect of nitrogen starvation, leading to the expression of subtelomeric genes, and this effect was suppressed by genetic deletion of *leo1*.

**Conclusions:**

We identified a protein, Leo1, necessary for survival during quiescence. Leo1 is part of a conserved protein complex, Paf1C, linked to RNA polymerase II. We showed that Leo1, acting downstream of TOR, is crucial for the dynamic reorganization of chromosomes and the regulation of gene expression during cellular quiescence. Genes encoding membrane transporters are not expressed in quiescent *leo1* mutant cells, and cells die after 2 weeks of nitrogen starvation. Taken together, our results suggest that Leo1 is essential for the dynamic regulation of heterochromatin and gene expression during cellular quiescence.

**Electronic supplementary material:**

The online version of this article (10.1186/s13072-019-0292-7) contains supplementary material, which is available to authorized users.

## Background

When fission yeast cells are starved for nitrogen, they rapidly divide twice without growth, arrest in the G1 phase of the cell cycle and enter cellular quiescence (G0). A gene regulatory program is initiated, including downregulation of thousands of genes—for example, genes related to cell proliferation—and upregulation of specific genes, such as autophagy genes, necessary for adaptation to the physiological challenge [1–3]. Thus, the transcriptional profile of quiescent *S. pombe* cells is dramatically reprogrammed to allow survival under low-nitrogen conditions [4]. This change is accompanied by a marked alteration of nuclear organization. The cell cycle arrest is reversible, and when nitrogen is added, quiescent cells readily exit the arrest and begin proliferating. Recently, two studies demonstrated that the RNA interference pathway is essential for viability during cellular quiescence. Dicer is important for heterochromatin assembly at centromeres during entry into G0 and for preventing heterochromatin formation over rDNA repeats in G0 cells [5]. Argonaute (Ago1), together with the H3K9 methyltransferase Clr4, plays a role in the repression of euchromatic genes in quiescent cells [6]. Together, the results of these studies clearly show that dynamic regulation of heterochromatin is essential during quiescence in fission yeast.

Leo1 is a subunit of the RNA polymerase-associated factor 1 (Paf1) complex (Paf1C). Paf1C consists of 5–6 subunits with four core subunits, including Leo1, which are conserved in eukaryotes. The complex has been implicated in several functions linked to RNA Pol II, including transcription elongation and mRNA 3’ end formation, and interacts with histone modifying enzymes, transcriptional activators, elongation factors and RNA cleavage factors [7]. A role for Paf1 in cell proliferation and terminal differentiation during development has been described in *Drosophila* [8]. Paf1C is also implicated in epigenetic regulation. For example, mutations in genes encoding Paf1 subunits enhance heterochromatin silencing by the RNAi pathway [9]. We previously found that two of the Paf1C core subunits, Leo1 and Paf1, are important for histone turnover and prevent heterochromatic regions from expanding in vegetative cells [10]. In this study, we addressed the role of Leo1 during cellular quiescence.

## Results

### Leo1 is required for survival during long-term quiescence

**Fig. 1:**
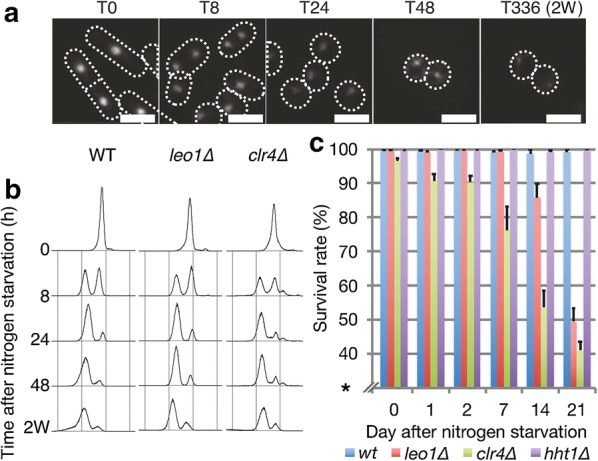
Leo1 is required for survival during long-term quiescence. **a** DAPI-stained images of wild-type cells at various time points during nitrogen starvation. Scale bars: 10 µm. **b** DNA content analysis by flow cytometry at various time points during nitrogen starvation. **c** Survival rate of the indicated strains at various time points during nitrogen starvation. The error bars represent the standard deviations (SDs) of three biological replicates

To identify additional factors involved in chromatin regulation during cellular quiescence, we applied a small-scale genetic screen using a selection of mutants affecting chromatin dynamics (Additional file 1: Fig. S1a). Of the twelve candidates tested, only *leo1∆* cells showed strongly reduced viability after long-term nitrogen starvation. After nitrogen starvation, *leo1∆* cells entered quiescence with kinetics similar to those of wild-type (WT) control cells (Fig. 1a, b); however, these cells exhibited a phenotype of reduced survival in G0. While the wild-type strain maintained a survival rate of 99.2% after 21 days in G0, the survival rate of *leo1∆* cells remained 99.4% at 7 days and then gradually decreased to 49.4% at 21 days (Fig. 1c). For comparison, the *clr4∆* cell survival rate was also decreased, in agreement with a previous report [6]. To investigate whether the other components of the Paf1C complex are also important during quiescence, we measured the survival rate of strains carrying genetic deletion of the Paf1C core components *leo1, paf1, cdc73, and tpr1 (ctr9)* during a 3-week quiescence time course experiment (Additional file 1: Fig. S1b, c). We also included the *prf1* mutant, but it was omitted from the survival analysis due to its peculiar cell morphology. The different mutations resulted in reduced survival of the host cells after 2–3 weeks of nitrogen starvation. Thus, Leo1 and three other core components of the Paf1C complex are required for survival during long-term quiescence.

### Leo1 affects the dynamics of heterochromatin assembly/disassembly at constitutive heterochromatic regions

**Fig. 2:**
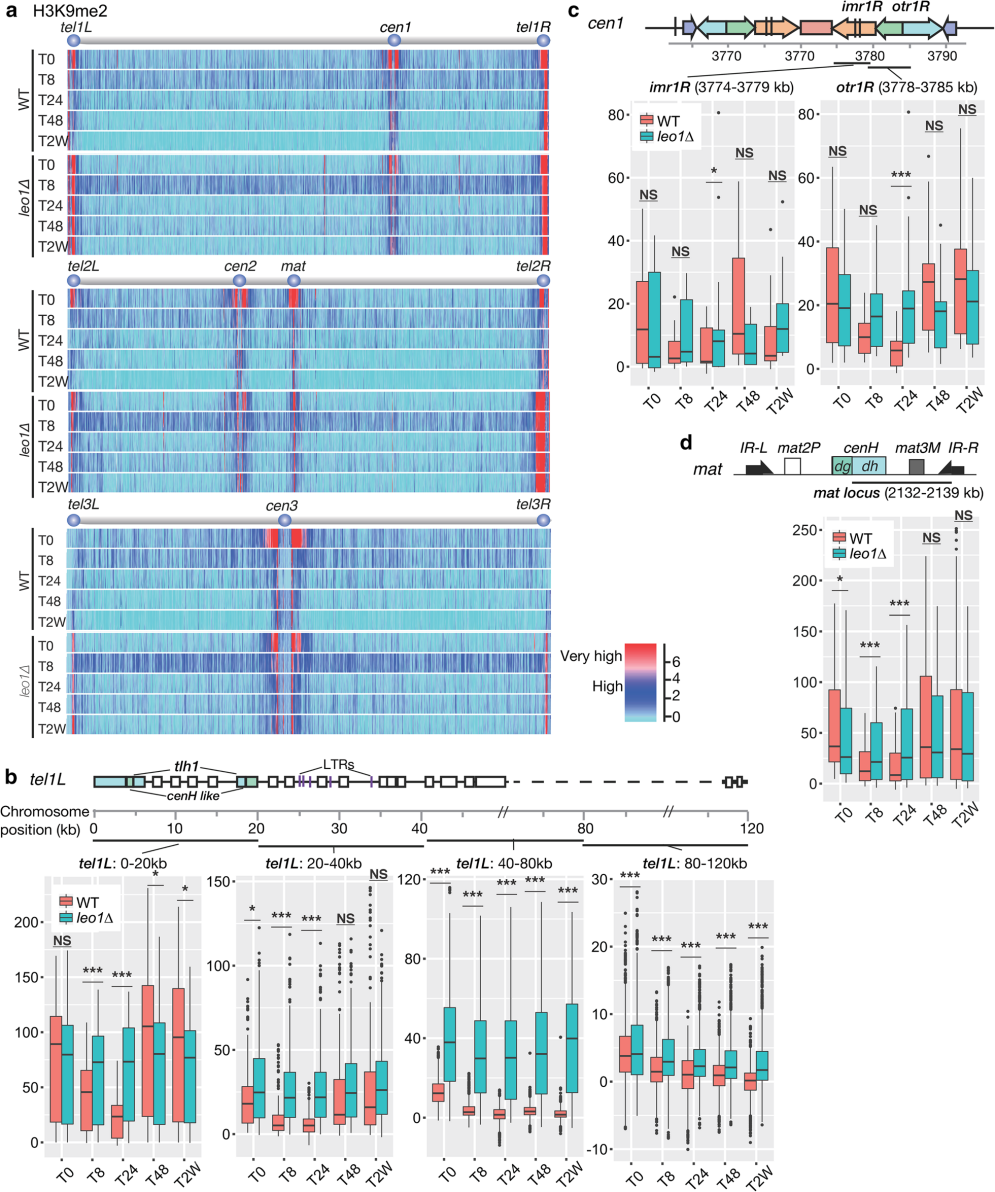
Loss of Leo1 causes mis-regulation of heterochromatin. **a** Mapping of H3K9 methylation in wild-type and *leo1∆* cells. The relative fold enrichment of di-methylated H3K9 (log2), as determined by ChIP-chip analysis with two biological replicates, is plotted. **b**–**d** Box plots showing changes in ChIP-chip signals for H3K9me2 at constitutive heterochromatin loci: the subtelomeric region *tel1L* (**b**); the pericentromeric region *cen1*; and the mating-type locus (**d**). The centre lines show the medians; the box limits indicate the 25th and 75th percentiles as determined by R software; the whiskers extend 1.5 times the interquartile range from the 25th and 75th percentiles, and outliers are shown. The y-axes show the relative enrichment of H3K9me2. The p values are from independent-samples *t*-test comparing the mean values between *leo1*∆ and WT at different time points. (NS) Non-significant; (*) *p* value < 0.05; (**) *p* value < 0.01; (***) *p* value < 0.001

We hypothesized that uncontrolled heterochromatin assembly in *leo1∆* cells limits the lifespan of these cells during long-term quiescence. To investigate this possibility, we performed genome-wide mapping of histone H3 di-methylation (H3K9me2) in wild-type and *leo1∆* cells during nitrogen starvation. Interestingly, in wild-type cells, the H3K9me2 levels in all heterochromatic regions that are constitutive in vegetative cells, i.e., centromeres, the mating-type region and telomere-proximal regions, diminished in early G0 phase (after 8 and 24 h) and were then partially restored after 48 h (see Fig. 2b, tel1L:0–20 kb; Additional file 1: Fig. S1). In sharp contrast, the H3K9me2 levels remained constant in *leo1∆* cells throughout G0 phase (Fig. 2; Additional file 2: Fig. S2). In subtelomeric regions of chromosomes I and II, the H3K9me2 levels in wild-type cells varied depending on the distance from the telomere and the time point during G0. In telomere-proximal regions (0–20 kb; 20–40 kb) wild-type H3K9me2 levels decreased at 8 and 24 h and then increased again at 48 h. In contrast, the subtelomeric H3K9me2 levels in *leo1∆* cells remained high throughout G0 phase (Fig. 2b; Additional file 2: Fig. S2). The wild-type H3K9me2 levels in pericentromeric regions (*imr* and *otr*) also varied depending on the time point in G0, whereas these levels in *leo1*∆ cells were much less dynamic (Fig. 2c; Additional file 2: Fig. S3). Finally, the same trends were observed in the mating-type region (*mat*) (Fig. 2d).

To test if the conversion of H3K9me2 to me3 is affected by *leo1Δ* cells we performed ChIP-QPCR for H3K9me3 at several heterochromatic loci at T0, T24 and T48 during quiescence (Additional file 3: Fig. S10). We find reduced H3K9m3e levels at all tested loci in wild-type cells at T24 and T48 compared to T0. This shows that the reduced H3K9me2 levels in quiescent cells are not due to conversion to H3K9me3. We also observe increased H3K9me3 levels in *leo1Δ* cells compared to wild type at all heterochromatic loci, especially at subtelomeric genes SPAC186.04c and SPBPB2B2.18 (Additional file 3: Fig. S10).

These observations show that constitutive heterochromatic regions in vegetative cells become subject to dynamic regulation during cellular quiescence leading to a transient reduction of both H3K9me2 and me3 and that Leo1 is required for this dynamic behavior of heterochromatin.

### Leo1 is critical for proper heterochromatin assembly at heterochromatin islands during G0 entry

In addition to subtelomeric heterochromatic regions, other H3K9me2-enriched regions were found in *leo1∆* cells relative to the H3K9me2 enrichment patterns in wild-type control cells (Additional file 2: Fig. S4). These regions overlapped with the previously identified islands of facultative heterochromatin, for example, at the *mcp7*^+^, *mei4*^+^, and *ssm4*^+^ loci [11]. The genes in these islands are regulated by the RNA elimination machinery (DSR) and the RNA processing complex (MTREC) [12]. In *leo1∆* cells, the H3K9me2 levels at these islands were approximately twofold higher than those in wild-type vegetative cells, decreasing gradually in early G0 and further diminishing after 2 weeks. These data suggest that Leo1 is critical for proper heterochromatin dynamics at DSR/MTREC-dependent heterochromatin islands during entry into G0 phase.

### Loss of Leo1 leads to the repression of genes located near the telomeres of chromosomes I and II

**Fig. 3:**
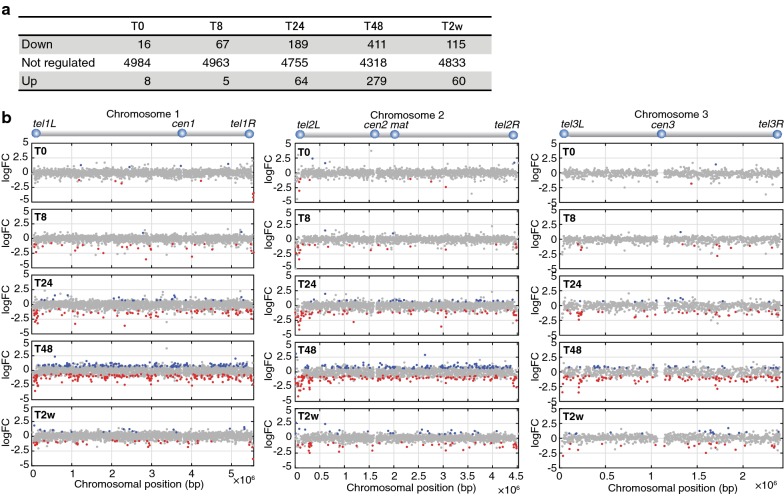
Loss of Leo1 leads to repressed transcription of genes located near the telomeres of chromosomes I and II. **a** The table shows the numbers of differentially regulated genes between *leo1∆* and wild-type cells at each time point during quiescence. The R/Bioconductor package EdgeR was used for the analysis of differential gene expression. For a gene to be deemed significant, the FDR adjusted p value had to be less than 0.05. ‘Down’, ‘Not changed’ and ‘Up’ indicate genes downregulated, unchanged and upregulated, respectively, in *leo1∆* cells relative to their expression in wild-type cells. Genes were filtered to remove genes with very low or no expression from the analysis. **b** Genome-wide distribution of regulated genes in *leo1∆* cells and wild-type cells. The *x*-axis indicates the chromosomal positions (× 10^−6^ bp), and the *y*-axis shows the log2 fold change (log2FC) values for genes differentially expressed between *leo1∆* and wild-type cells. Up- and downregulated genes are shown in blue and red, respectively (FDR < 0.05)

Genome-wide mapping of H3K9me2 showed that Leo1 counteracts heterochromatin assembly, which is normally associated with transcriptional repression. To determine whether Leo1 affects gene expression in G0, we performed RNA sequencing (RNA-seq) analysis in wild-type and *leo1∆* cells after the shift to nitrogen-free medium. As expected, the expression of many genes was lower in *leo1∆* mutant cells than in wild-type cells, especially at 24 and 48 h after the shift (Fig. 3a). When plotted along the chromosomes, the downregulated genes were enriched near the telomeres of chromosomes I and II (Fig. 3b and Additional file 2: Fig. S5). In contrast, the *leo1∆* mutation did not affect gene expression at the ends of chromosome III, which containing rDNA repeats. To investigate the statistical significance of telomere proximity for the genes downregulated in *leo1∆* versus wild-type cells, we performed a Chi-square test (Additional file 2: Fig. S8), which revealed that the downregulated genes were significantly clustered in subtelomeric regions 0–80 kb from the telomere at *tel1L, tel1R, tel2L* and *tel2R*. This effect was observed starting 8 h after nitrogen removal and persisted throughout the 2-week quiescence time course. Thus, these results clearly show that loss of Leo1 leads to the repression of genes throughout the genome, with a stronger effect for genes near the telomeres of chromosomes I and II.

### Loss of Leo1 prevents the expression of membrane transporter-encoding genes in G0

**Fig. 4:**
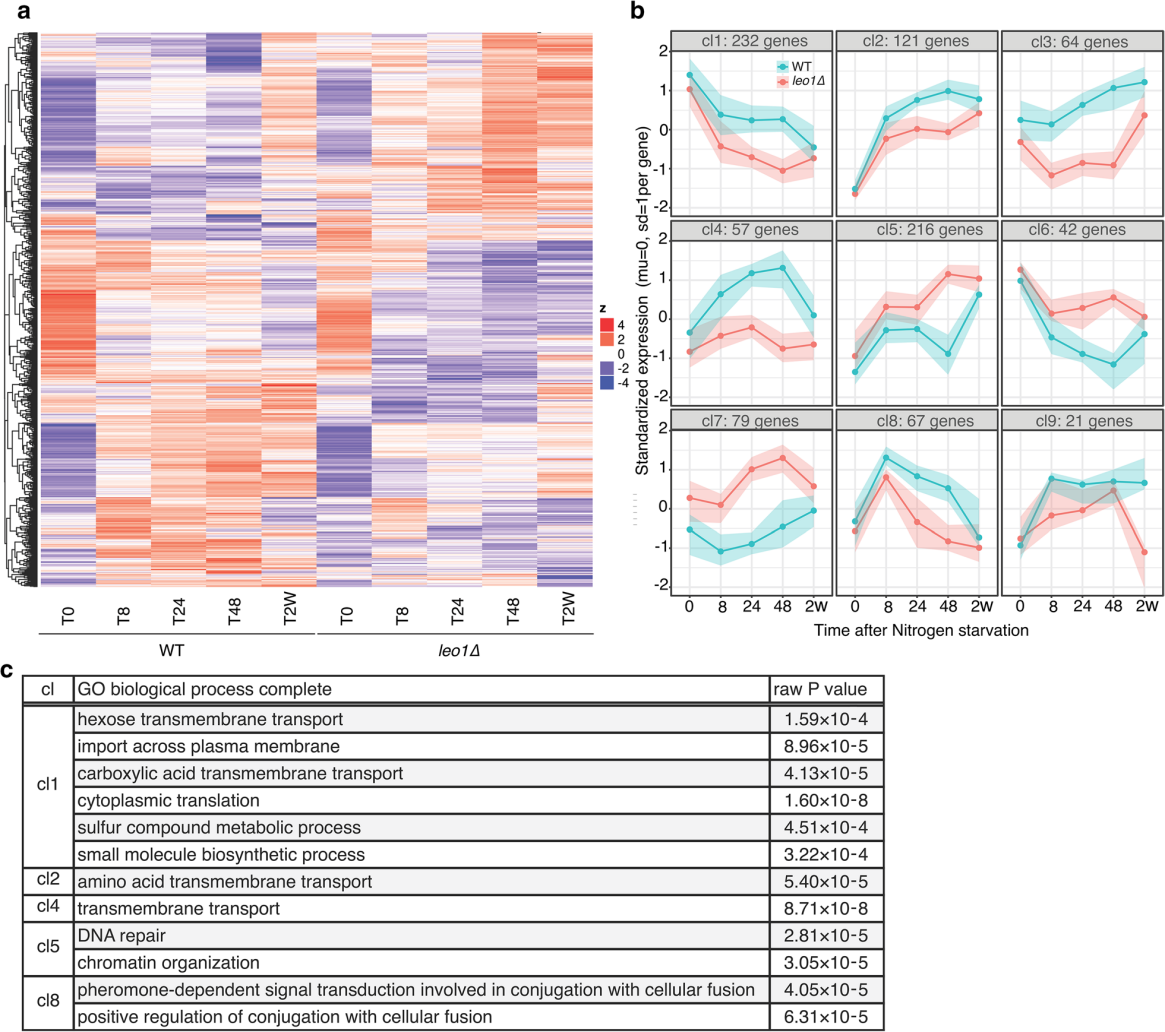
Loss of Leo1 causes the downregulation of membrane transporter-encoding genes. **a** A heatmap of all the genes found to be significant in any of the comparisons shown in Fig. 3. The expression of each gene was standardized (mean = 0, SD = 1), and the genes were then clustered by hierarchical clustering. **b** The clustering performed for the heatmap was limited to 9 clusters and plotted as lines to enhance the visual representation of the gene regulation patterns. The lines indicate the medians of the cluster, and the colored areas surrounding the lines show the 25th and 75th percentiles, meaning that 50% of the genes were in the cluster. **c** List of the GO biological process terms enriched with the differentially expressed genes in the clusters. The significance of the GO terms was determined using the FDR correction (*p *< 0.05). The granular terms with the largest fold enrichment are shown for each group. Clusters cl3, cl6, cl7 and cl9 had no apparent GO term enrichment

Next, we analyzed the expression patterns of all differentially expressed genes using hierarchical clustering. The differentially expressed genes clustered into nine major clusters (cl) based on the patterns of expression at different time points. The clusters are shown on a heat map and in a graphical format (Fig. 4a, b). Four clusters exhibited lower expression in *leo1∆* cells than in wild-type cells, with decreasing (cl1) or increasing (cl2, 3 and 4) expression levels during early G0. Three clusters exhibited higher expression in *leo1∆* cells, with increasing (cl5 and 7) or decreasing (cl6) expression levels during early G0. The final two clusters were mirror images, with a peak at T8 (cl8) or T48 (cl9).

To gain insight into the biological processes regulated during quiescence, we performed Gene Ontology (GO) analysis of differentially expressed genes within these nine clusters and identified the associated enriched GO terms (Fig. 4c). In clusters 1, 2, and 4, which showed lower expression in *leo1∆* cells than in wild-type cells during quiescence, GO terms for the processes of trans-membrane transport of hexose and amino acids were significantly enriched (FDR < 0.01). In cluster 5, which showed higher expression in *leo1∆* cells during quiescence, the GO terms for the processes of DNA repair and chromatin organization were significantly enriched. Finally, for cluster 8, with a peak at T8, the GO term for conjugation with cellular fusion was significantly enriched.

### Paf1C stimulates heterochromatic histone turnover in G0 cells

**Fig. 5:**
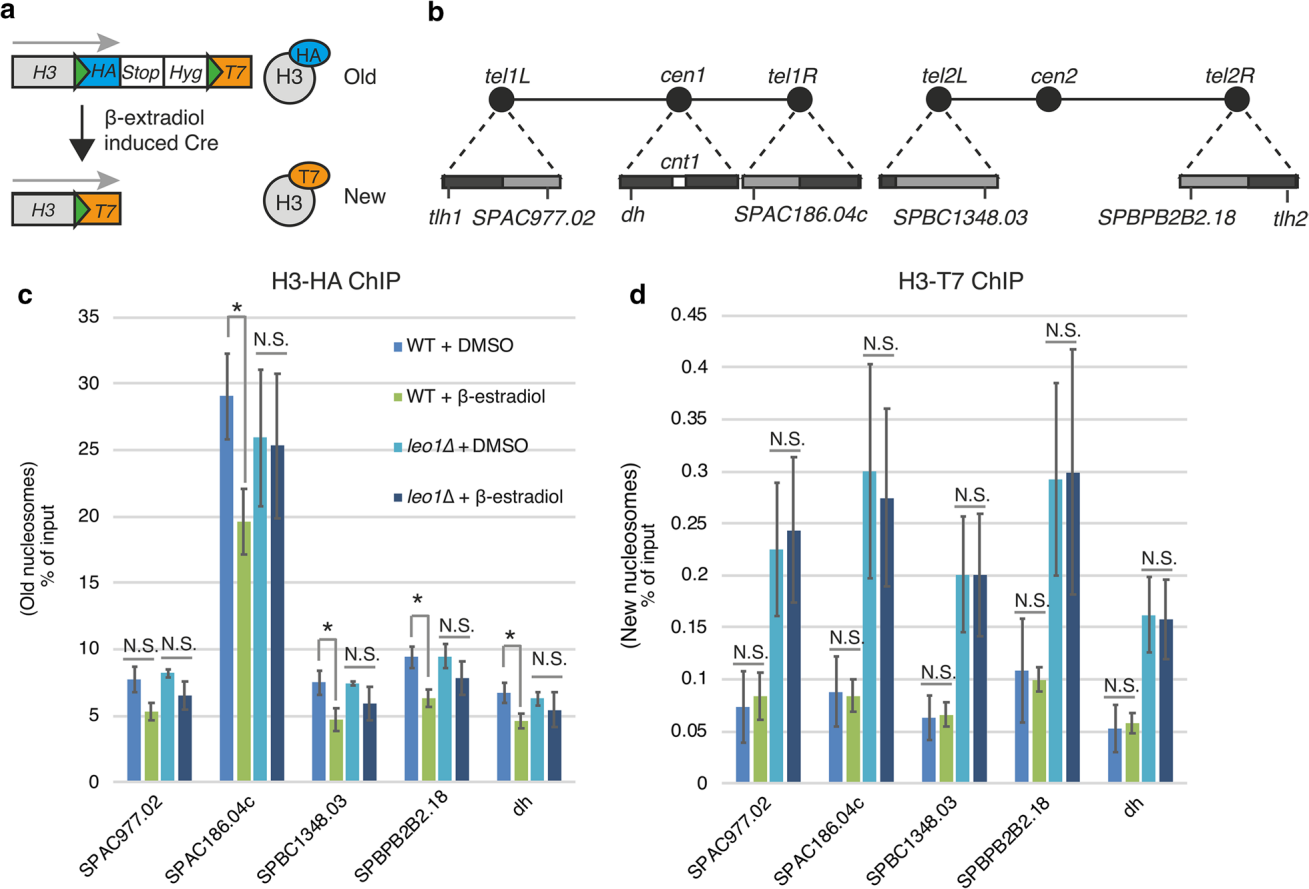
Leo1 regulates histone eviction during quiescence. **a** Diagram of the RITE system. The green triangles represent *LoxP* sites. **b** A schematic of fission yeast chromosomes I and II. The triangles on the chromosomal edges are enlarged views of centromeric and subtelomeric regions. The dark and light gray boxes indicate regions of high and low H3K9me2, respectively. ChIP of H3-HA (**c**) and T7-HA (**d**) in silent chromatin regions. The values are the averages of three biological replicates. The error bars indicate SDs; *p* values (paired Student’s *t* test): *< 0.05; N.S. (not significant) > 0.05

We previously showed that the turnover of histone H3 in heterochromatic regions of fission yeast is reduced in vegetative cells lacking the Leo1 subunit of Paf1C [10]. To investigate whether histone turnover was affected in G0 cells, we performed recombination-induced tag exchange for histone H3 (H3-RITE) experiments (Fig. 5a). For this analysis, we focused on a set of four subtelomeric genes repressed in vegetative cells by Paf1C [13] (Fig. 5b). First, we measured the recombination rates by scoring the frequency of hygromycin-sensitive colonies. At T0, after 2 h of Cre recombinase induction, wild-type and *leo1∆* cells exhibited recombination rates of 48.1% and 49.4%, respectively. At T24, after 4 h of Cre recombinase induction, wild-type and *leo1∆* cells exhibited recombination rates of 35.4% and 35.5%, respectively. Next, we measured histone exchange but found no significant increase in T7-tagged histones after tag exchange, suggesting that the incorporation of new histones is very slow in quiescent cells. However, we did observe reduced levels of old (HA-tagged) histones in wild-type cells after 24 h in G0 (Fig. 5c). This reduction was dependent on Leo1, since the level of old histones was not significantly reduced in *leo1∆* cells. Thus, we concluded that Paf1C stimulates the eviction of old histones in G0 cells.

### The activity of the TORC2/Gad8 pathway is modulated during quiescence

**Fig. 6:**
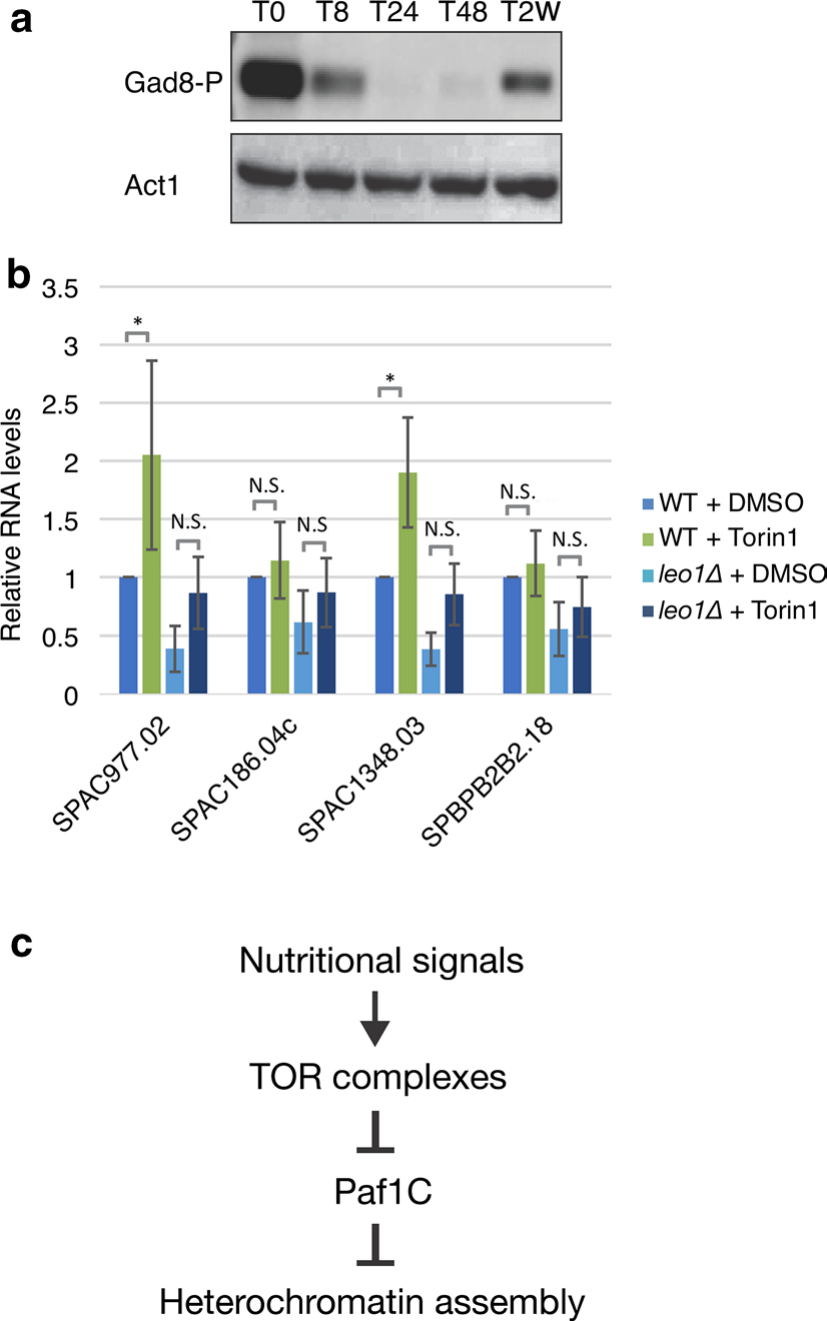
The Tor1/Gad8 pathway regulates Paf1C. **a** Phosphorylation of Gad8 at various time points after nitrogen starvation. **b** RNA expression levels of subtelomeric genes on chromosome I and II were quantified by RT-qPCR. Cells were treated with DMSO or 25 µM Torin1. Human RNA was used as the spike-in control RNA for normalization of the expression levels. The values are the averages of three biological replicates (n = 6). The error bars indicate SDs; *p* values (paired Student’s *t* test): *< 0.05; N.S. (not significant) > 0.05. **c** A proposed model for the regulation of heterochromatin via TOR complexes and Paf1C in G0 cells (see the main text for details)

To investigate the means by which Leo1 regulates heterochromatin during quiescence, we focused on target of rapamycin (TOR) complex 2 (TORC2). TORC2 responds to glucose levels, and because it activates the protein kinase Gad8 (an orthologue of human AKT), it is required for the regulation of cell cycle progression, starvation responses, and cell survival [14–16]. In addition to these functions, TORC2 was recently reported to be required for gene silencing and the formation of heterochromatin in fission yeast. Tor1 and Gad8 are needed for the silencing of subtelomeric heterochromatic regions [13]. The function of Tor1/Gad8 is linked to Paf1C, since the silencing defects in subtelomeric regions in *tor1∆* and *gad8∆* mutant cells are fully suppressed by genetic deletion of *paf1* [13]. Based on this finding, we hypothesized that a decrease in TORC2 activity could induce the transient reduction in H3K9me2 levels at subtelomeric regions at 24 h after nitrogen starvation. Therefore, we monitored the protein level of activated Gad8 during quiescence. Western blotting was carried out using specific antibodies against the active phosphorylated form of the Gad8 kinase (Fig. 6a; Additional file 2: Fig. S9). Although some variation between biological duplicates was observed, all results showed a similar trend: the level of activated Gad8 clearly diminished in early G0 phase (after 8 and 24 h) and was subsequently restored after 48 h and 2 weeks. These changes in Gad8 activity were inversely correlated with the observed Leo1-dependent changes in H3K9me2 in heterochromatic regions during G0 phase (Fig. 2; Additional file 2: Figs. S2, S3). Moreover, we found no change in Gad8 activation between *leo1∆* cells and wild-type cells (Additional file 2: Fig. S9), suggesting that Gad8 acts upstream of Paf1C.

To examine the effect of TOR activity on the expression of subtelomeric genes, we used the TOR inhibitor Torin1 and studied gene expression by RT-PCR using the same set of four genes (Fig. 6b). Torin1 treatment clearly mimicked nitrogen starvation conditions, leading to a significant increase in the expression of these genes (Fig. 6b). However, treatment of *leo1∆* cells with Torin1 did not lead to significant upregulation (Fig. 6b). Therefore, we propose that Paf1C activity in G0 cells is dependent on TOR complexes (Fig. 6c).

## Discussion

### A mechanism of chromatin dynamics involving the Leo1 subunit

Interestingly, the structure of Paf1C in complex with Pol II reveals that the Paf1 and Leo1 subunits are positioned in front of the Pol II complex near the downstream DNA in a perfect position to meet the incoming nucleosomes during elongation [17]. Therefore, this domain of Paf1C is likely positioned appropriately for involvement in nucleosome disassembly during transcription, allowing the turnover of histones. Here, we observed reduced levels of old histones in G0 cells, and this reduction was dependent on Leo1.

Previous work showed that the turnover of histone H3 in heterochromatic regions of vegetative cells is also dependent on Leo1 [10]. H3K9me2 regions have recently been shown to be permissive for transcription in fission yeast [18]. It is plausible that the increased stability of heterochromatin in quiescent *leo1Δ* cells is caused by a failure to remove old histone H3, thereby stabilizing the H3K9me2 level. We previously showed that Leo1 counteracts H3K9me2 in vegetative cells [10]. Here, we show that some heterochromatic regions are destabilized in wild-type cells and that H3K9me2 levels are reduced during quiescence in a Leo1-dependent manner. Deletion of the *leo1* gene leads to both up- and downregulation of genes relative to their wild-type levels during quiescence (Fig. 3a). To further explore the effects of *leo1* gene deletion on gene expression, we directly compared changes in RNA expression levels with changes in H3K9me2 levels (Additional file 2: Figs. S6, S7). This analysis revealed a propensity for downregulated genes to be located in regions with increased H3K9me2 levels in *leo1∆* cells, suggesting that direct stabilization of heterochromatin leads to gene repression. Quiescent cells are arrested in the G0 phase of the cell cycle and do not undergo DNA replication, which, in vegetative cells, contributes to histone turnover at the replication fork. Therefore, Paf1C activity may be especially important in G0 cells to remove old histones marked by H3K9me2 in order to allow epigenetic reprogramming to support the gene expression changes needed for adaptation to the low-nitrogen environment.

### Changes in subtelomeric gene expression mediated by Paf1C

We observed decreased expression of genes involved in the transport of hexose and amino acids in *leo1∆* cells during G0. This decrease was accompanied by an expansion of heterochromatin in the subtelomeric regions where many of these genes are located. These findings strongly suggest that Paf1C has a special role in subtelomeric heterochromatic regions to allow the reduction of heterochromatin and the induction of gene expression in these regions to adapt to cellular quiescence. Genes in the subtelomeric regions showed Leo1-dependent histone turnover (Fig. 5c) and had lower expression levels in *leo1∆* cells than in wild-type cells (Fig. 6b). Analysis of the time course data showed that downregulation started 8 h after the nitrogen shift and that the genes remained repressed throughout the 2-week time course (Additional file 2: Fig. S8). Since several of the subtelomeric genes encode transporters, the reduced viability of *leo1∆* cells observed after 2–3 weeks in G0 may be caused by transport deficiencies due to failure to express these genes. The phenotypic lag period between the reduced expression and loss of viability in G0 may be due to reductions in the levels of nutrients that become critical after 2 weeks.

### Is the function of Paf1 in epigenome dynamics conserved?

A role for the Paf1C component Ctr9 in cell differentiation has recently been reported in *Drosophila* [8]. *Drosophila* neurons fail to terminally differentiate, and hundreds of genes, including several neuropeptide-encoding genes, are downregulated in *ctr9* mutants. A reduction in the activating histone mark H3K4me3 was observed in *ctr9* mutants [8]. However, whether H3K9 methylation is increased in *Drosophila ctr9* mutants in the same way as in *leo1* mutant fission yeast is unknown, and whether Paf1C is important for the heterochromatin changes that occur during cell differentiation and quiescence in other species remains to be investigated.

### A model linking the functions of TOR complexes and Paf1C during quiescence

The observed reduction in TORC2 activity could explain the reduced H3K9me2 levels in heterochromatic regions in G0 cells. TORC2 was recently shown to be required for subtelomeric silencing and heterochromatin assembly in vegetative cells [13]. The Gad8 kinase is present in the nucleus [19]; therefore, it is therefore conceivable that the TORC2/Gad8 pathway directly regulates Paf1C during quiescence. We showed that Torin1 treatment de-repressed some subtelomeric genes but did not affect *leo1∆* cells (Fig. 6a, b). Torin1 inhibits both the TORC1 and TORC2 complexes in fission yeast [20]. Therefore, both TOR complexes possibly act upstream of Paf1C in the control of heterochromatin dynamics in quiescent cells (Fig. 6c).

## Conclusions

Here, we demonstrated two important concepts:

1. During cellular quiescence in fission yeast, heterochromatic regions become very dynamic, especially in subtelomeres.
2. Paf1C, acting downstream of TOR complexes, is a key player in the dynamic regulation of heterochromatin in quiescent cells, allowing changes in gene expression and adaptation to the new cellular environment.

Paf1C is conserved in eukaryotes [7], and a component of Paf1C was recently implicated in a cell differentiation process in *Drosophila* [8]. The dynamic regulation of heterochromatin in quiescent cells by Paf1C may also contribute to the function of Paf1C in cell differentiation in multicellular eukaryotes.

## Methods

### Yeast strains and growth conditions

A selection of strains from the Bioneer library version 5 carrying deletions of genes encoding various chromatin regulators was backcrossed, *mat1*-*M smt*-*0* strains carrying gene deletions marked by *kanMX* were selected, and the mating type was confirmed by PCR [21]. The following strains were used for further studies: PB1623 *mat1*-*M smt*-*0*; PB2423 *mat1*-*M smt*-*0 leo1∆*; PB2426 *mat1*-*M smt*-*0 hht1∆*; and PB2428 *mat1*-*M smt*-*0 clr4∆*. For liquid cultures, pombe minimal glutamate (PMG) and PMG without nitrogen (PMG-N) media were used. For the genetic screen, survival during long-term quiescence was followed in 20-ml PMG-N cultures at 32 °C for 48 days by plating aliquots of cells on rich medium (yeast extract with supplements, YES) at regular intervals.

### Quiescence induction

Cells were grown in PMG medium at 30 °C to a density of OD_600_ ~ 0.25. Quiescence was induced by washing cells twice with PMG-N medium and resuspending them in an equal volume of PMG-N medium.

### Propidium iodide (PI) staining and flow cytometry

Cells grown in PMG or PMG-N medium were collected and fixed with 70% ethanol. Then, cells were washed with 50 mM sodium citrate (pH 7.0) and treated with 240 µg/ml RNase A in 50 mM sodium citrate (pH 7.0) for 1 h at 37 °C. Cells were stained with 1.25 µg/ml PI in 50 mM sodium citrate (pH 7.0). Stained cells were imaged with a confocal microscope using the Cy5 channel. The DNA content was monitored using a FACSCalibur (Becton Dickinson). Data analysis was carried out with Cell Quest software.

### Viability assay

Viability assays were performed according to the protocol described in Joh et al. [6], with minor modifications. A 1 ml aliquot of cell suspensions was taken from incubated cell cultures at the indicated time points and stained with Phloxin B (5 µg/ml final concentration) for 15 min. Cells were washed with 1 × phosphate-buffered saline (PBS) and imaged with an Axioplan 2 fluorescence microscope and ImageJ software using the FITC and DIC channels. A minimum of 400 cells was counted for each measurement using ImageJ software, and survival rates were calculated based on the number of dead (stained and green) cells divided by the total number of cells (green and stain-free) counted.

### Chromatin immunoprecipitation (ChIP) analysis

Chromatin was extracted from cultures in duplicate and subjected to immunoprecipitation. Samples were immediately subjected to formaldehyde cross-linking (final concentration 1%), and chromatin was isolated as described by Durand-Dubief et al [22]. ChIP was performed using 4 µg of anti-H3K9me2 antibodies (Abcam, ab1220) per 30 µl of chromatin extract. DNA was recovered with QIAquick PCR Purification columns (Qiagen).

### ChIP microarray

For microarray hybridization, immunoprecipitated DNA was amplified as described by Durand-Dubief et al [22]. Fragmentation, labeling and hybridization to the Affymetrix GeneChip *S. pombe* Tiling 1.0FR were performed by the BEA core facility at Novum (http://www.bea.ki.se) according to Affymetrix standard protocols. For domainogram visualization, raw data from Affymetrix (.CEL format) were normalized using Affymetrix Tiling Analysis Software (TAS) v1.1. Duplicate data were normalized to input using quantile normalization plus scaling using a bandwidth of 100. Data obtained from TAS were then directly loaded into the Seqmonk program, and the antibody background was removed using data for the *clr4∆* mutant. Data were visualized using the following parameters: number of classes, 30; min probe count, 30; and max probe count, 100. Raw and normalized ChIP microarray data have been submitted to the Gene Expression Omnibus under accession number GSE116038.

### RNA isolation

Cells were harvested by centrifugation at 3000 rpm and 25 °C. Then, cells were washed one time with PBS (pH 7.4). The pellet was re-suspended in 500 µl of RNA extraction buffer (2% Triton X-100, 1% SDS, 100 mM NaCl, 10 mM Tris–HCl (pH 8.0), and 1 mM EDTA (pH 8.0)) and 500 µl of acid phenol (pH 5.2). Then, 500 µl of acid-washed glass beads were added to the tubes. The tubes were vortexed vigorously at 4 °C to disrupt the cells. The tubes were centrifuged at 13,000 rpm for 5 min at 4 °C, and the upper aqueous phase was collected into a gel phase tube containing an equal volume of chloroform. After mixing, the tubes were centrifuged at 13,000 rpm for 5 min at 25 °C, and the upper aqueous phase was collected. Ammonium acetate (pH 5.2) was added to a final concentration of 2.5 M, along with 2.5 volumes of ethanol. The tubes were stored at − 20 °C overnight for RNA precipitation. The precipitated RNA was washed one time with 70% ethanol and dissolved in RNase-free water. DNase I treatment was performed using TURBO DNase (Thermo Fisher Sci.) following the manufacturer’s instructions.

### RNA-seq

To remove rRNA, 4 µg of purified total RNA was treated with a ScriptSeq Complete Gold Kit (Yeast) (Epicentre). A total of 40 ng of rRNA-depleted stocks were used to generate sequencing libraries using a ScriptSeq v2 RNA-seq Library Preparation Kit (Epicentre). Samples were quantified using a Qubit (HS dsDNA) and sequenced using an Illumina HiSeq 2000 platform (50 cycles, single-end sequencing) at the BEA facility (Huddinge, Sweden) following the manufacturer’s instructions. The raw HiSeq data (fastq files) were aligned to ASM294v2 using Bowtie2 with the default parameters. The ASM294v2.24 annotation was downloaded from pombase.org. The aligned data (SAM files) were imported and normalized per million reads. Data from independent biological duplicates were averaged. Identical reads were discarded to remove PCR artefacts. Signals were calculated as averages over 150 nt.

### H3-rite

H3-RITE was carried out according to the procedure described in [10] except for the changes in the growth media. Here, cells were cultured in PMG-N medium at 30 °C for 24 h after the change from PMG medium. To induce the genetic switch, cells were treated with 1 µM β-oestradiol for 4 h at 30 °C.

### Torin1 treatment

Cells were grown in PMG-N medium at 28 °C to a density of OD_600_ ~ 0.25 and treated with Torin1 for 30 min at a final concentration of 25 µM or with an equivalent volume of DMSO vehicle control [23].

### RT-PCR

For RT-PCR analysis, total RNA was cleaned and treated with TURBO DNase (Thermo Fisher Scientific) according to the manufacturer’s instructions. RT-PCR was performed using a SuperScript III First-Strand Synthesis System for RT-PCR (Thermo Fisher Scientific) according to the manufacturer’s instructions. For RT-PCR, 400 ng of total RNA from *S. pombe* was used per reaction, and 50 ng of human RNA extracted from human bone osteosarcoma epithelial cells (U2OS cell line) was added as the spike-in control RNA for normalization of the expression levels.

### qPCR

qPCR was performed using FastStart Universal SYBR Green Master (Rox) (Sigma-Aldrich) in a 7500 Fast Real-Time PCR System (Applied Biosystems). For RT-qPCR, human GAPDH levels were measured for the normalization of RNA levels. The primer sequences used are shown in Table S1.

### Western blotting

Protein was extracted from vegetative and quiescent cells. A total of 20 µg of protein was separated by 10% SDS–PAGE and transferred to nitrocellulose membranes. Membranes were incubated with anti-phosphorylated Gad8 (custom-made) (1:1000) and anti-actin (MP #691,001) (1:1000) antibodies, and immunoreactions were detected by an ECL (SuperSignal Detection System, Thermo Scientific) kit.

## Additional files

**Additional file 1.** Supplementary data Figure S1.

**Additional file 2.** Supplementary data Figures S2–S9.

**Additional file 3.** Supplementary data Figure S10.

## Data Availability

Genome-wide data have been submitted to the Gene Expression Omnibus under the accession number GSE116038.

## Abbreviations

TOR: target of rapamycin
TORC2: target of rapamycin complex 2
Paf1C RNA: polymerase-associated factor 1 complex
Ago: argonaute
H3-RITE: recombination-induced tag exchange for histone H3
PMG: pombe minimal glutamate medium
