# Supplementary material for: Leo1 is essential for the dynamic regulation of heterochromatin and gene expression during cellular quiescence
Source: Epigenetics Chromatin. 2019 Jul 17;12:45. doi: 10.1186/s13072-019-0292-7 (PMC6636030; doi:10.1186/s13072-019-0292-7)
Supplement: Supplementary file 1 — Additional file 1. Supplementary data Figure S1. [file 13072_2019_292_MOESM1_ESM.pptx]

## Slide 1
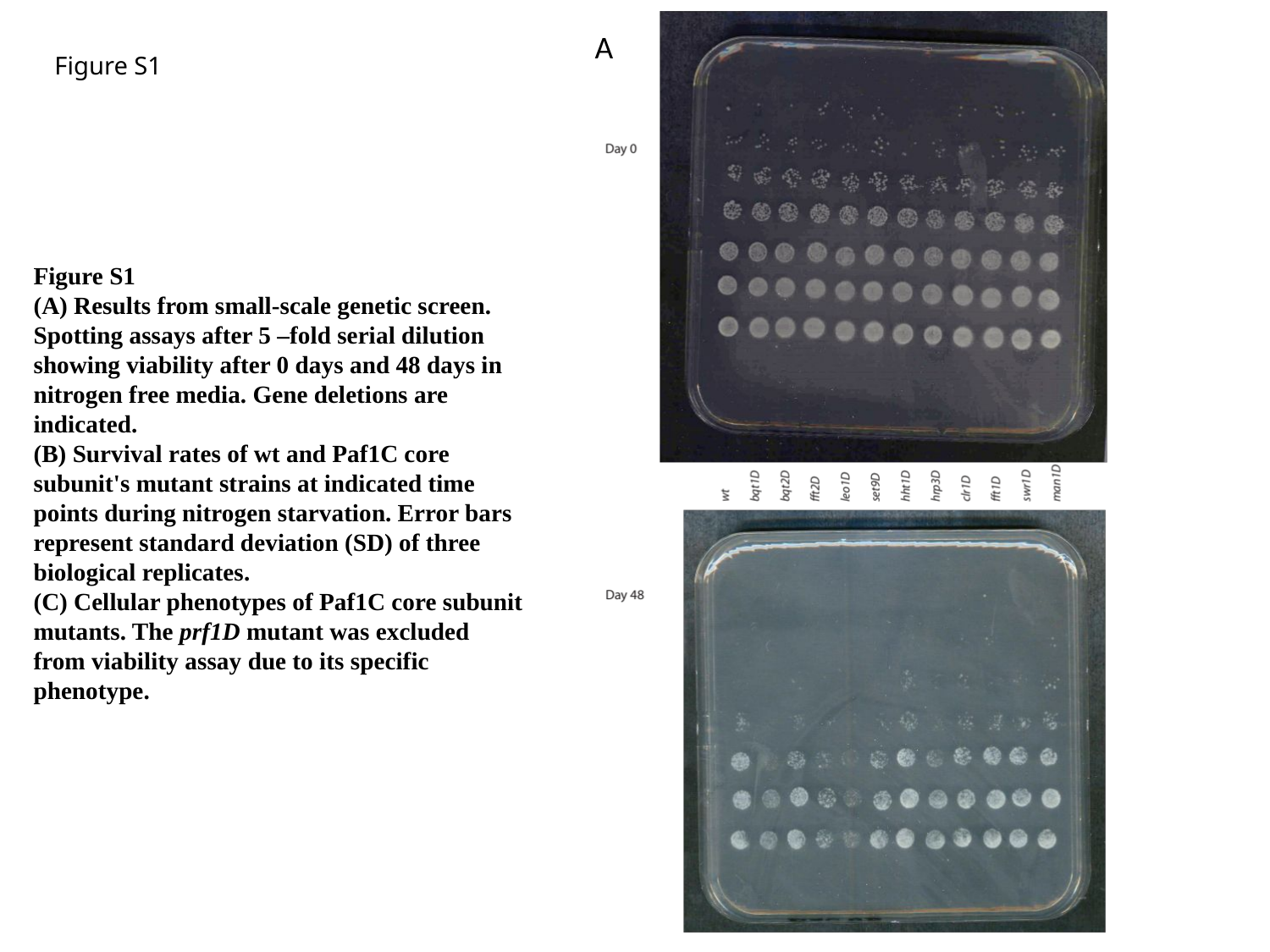

A
Figure S1
Figure S1
(A) Results from small-scale genetic screen. Spotting assays after 5 –fold serial dilution showing viability after 0 days and 48 days in nitrogen free media. Gene deletions are indicated.
(B) Survival rates of wt and Paf1C core subunit's mutant strains at indicated time points during nitrogen starvation. Error bars represent standard deviation (SD) of three biological replicates.
(C) Cellular phenotypes of Paf1C core subunit mutants. The prf1D mutant was excluded from viability assay due to its specific phenotype.

## Slide 2
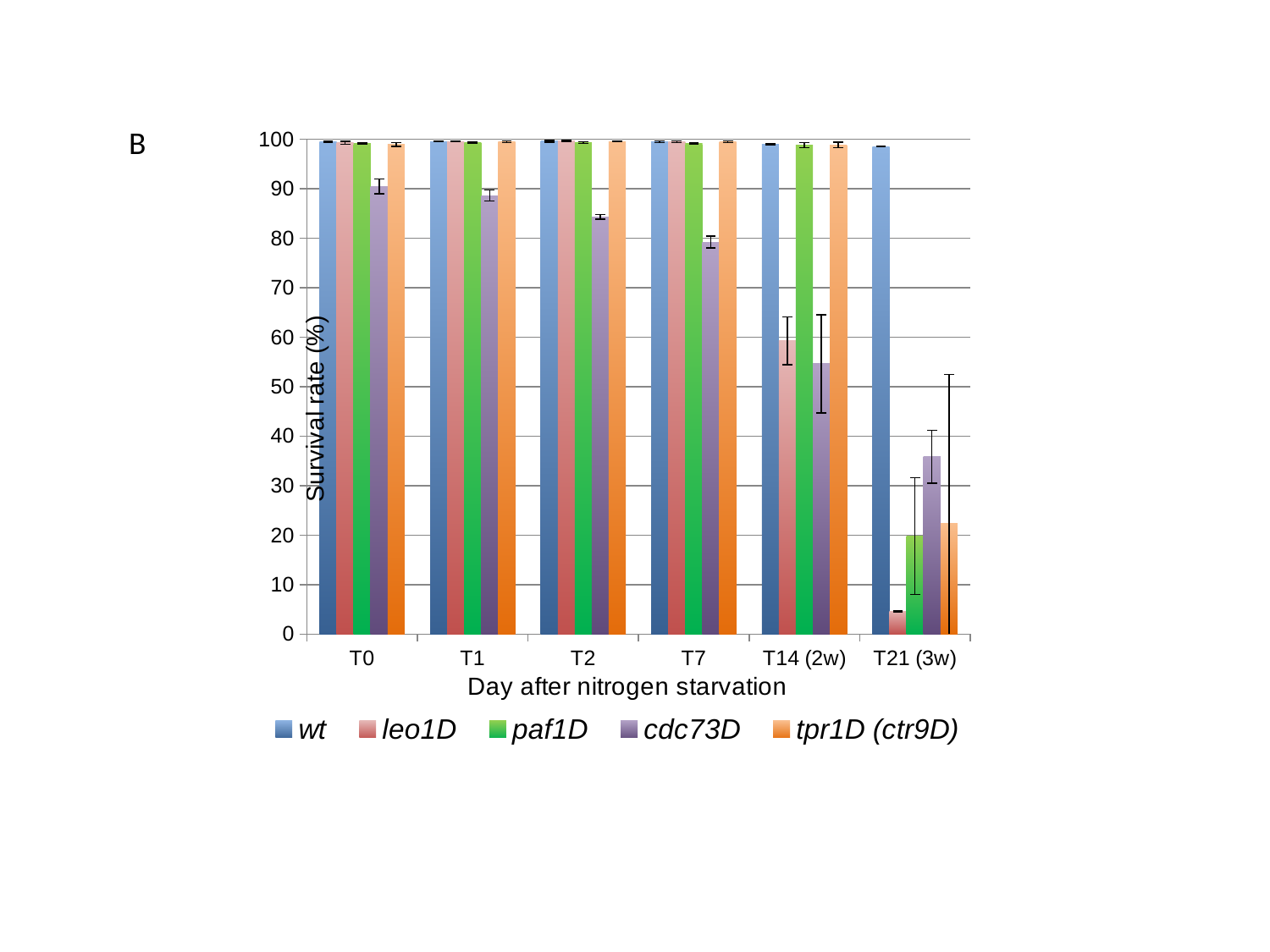

B
### Chart
| Category | wt | leo1D | paf1D | cdc73D | tpr1D (ctr9D) |
|---|---|---|---|---|---|
| T0 | 99.45094234910233 | 99.24469755868965 | 99.12002608348568 | 90.45387300237411 | 98.9430304650691 |
| T1 | 99.55876104588828 | 99.55291203215155 | 99.31320432000393 | 88.62179109652526 | 99.4942810571804 |
| T2 | 99.57595376703877 | 99.62915448360128 | 99.29531439637307 | 84.31735048255848 | 99.52995780941838 |
| T7 | 99.4999150428633 | 99.46767289470435 | 99.14111581721158 | 79.22713314100277 | 99.49639112811053 |
| T14 (2w) | 99.02582777779338 | 59.28356115556507 | 98.80634192050343 | 54.61170224061345 | 98.85385252398468 |
| T21 (3w) | 98.51542651700258 | 4.519542449624701 | 19.8365576891107 | 35.83730217196964 | 22.34600471230251 |

## Slide 3
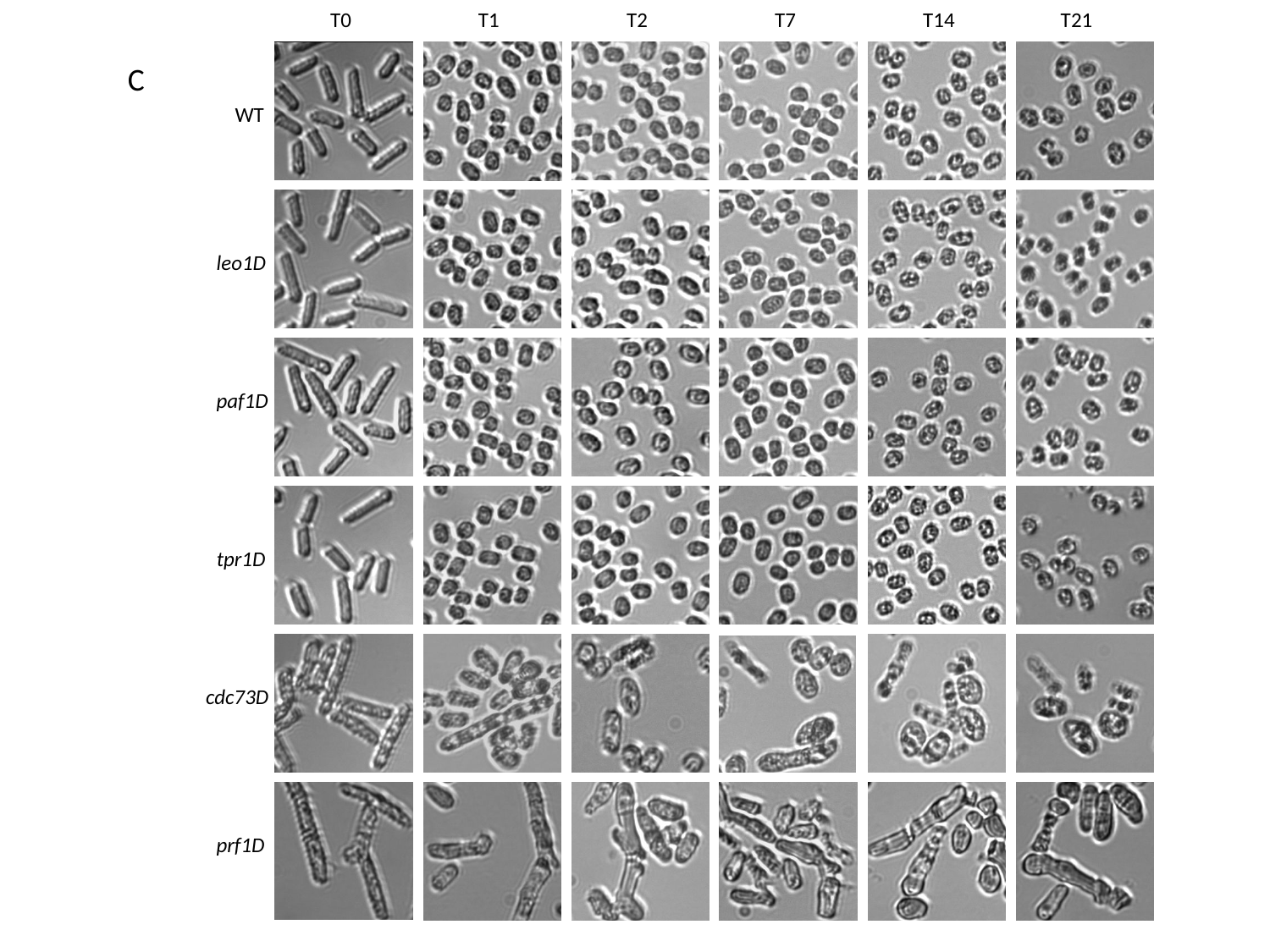

T0
T1
T2
T7
T14
T21
C
WT
leo1D
paf1D
tpr1D
cdc73D
prf1D
