# Supplementary material for: Leo1 is essential for the dynamic regulation of heterochromatin and gene expression during cellular quiescence
Source: Epigenetics Chromatin. 2019 Jul 17;12:45. doi: 10.1186/s13072-019-0292-7 (PMC6636030; doi:10.1186/s13072-019-0292-7)
Supplement: Supplementary file 2 — Additional file 2. Supplementary data Figures S2–S9. [file 13072_2019_292_MOESM2_ESM.pptx]

## Slide 1
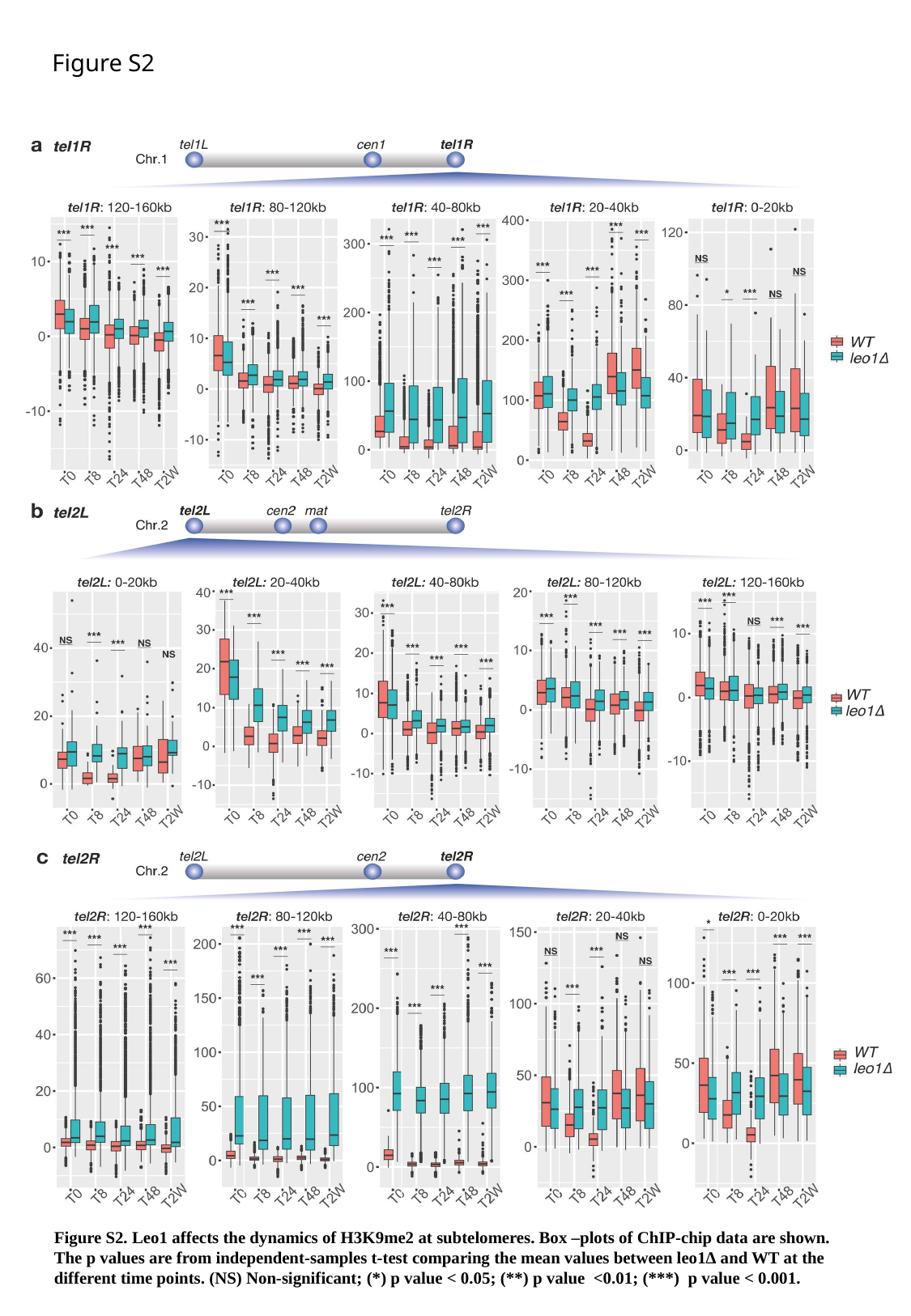

Figure S2
Figure S2. Leo1 affects the dynamics of heterochromatin assembly at subtelomeres.
Figure S2. Leo1 affects the dynamics of H3K9me2 at subtelomeres. Box –plots of ChIP-chip data are shown. The p values are from independent-samples t-test comparing the mean values between leo1∆ and WT at the different time points. (NS) Non-significant; (*) p value < 0.05; (**) p value <0.01; (***) p value < 0.001.

## Slide 2
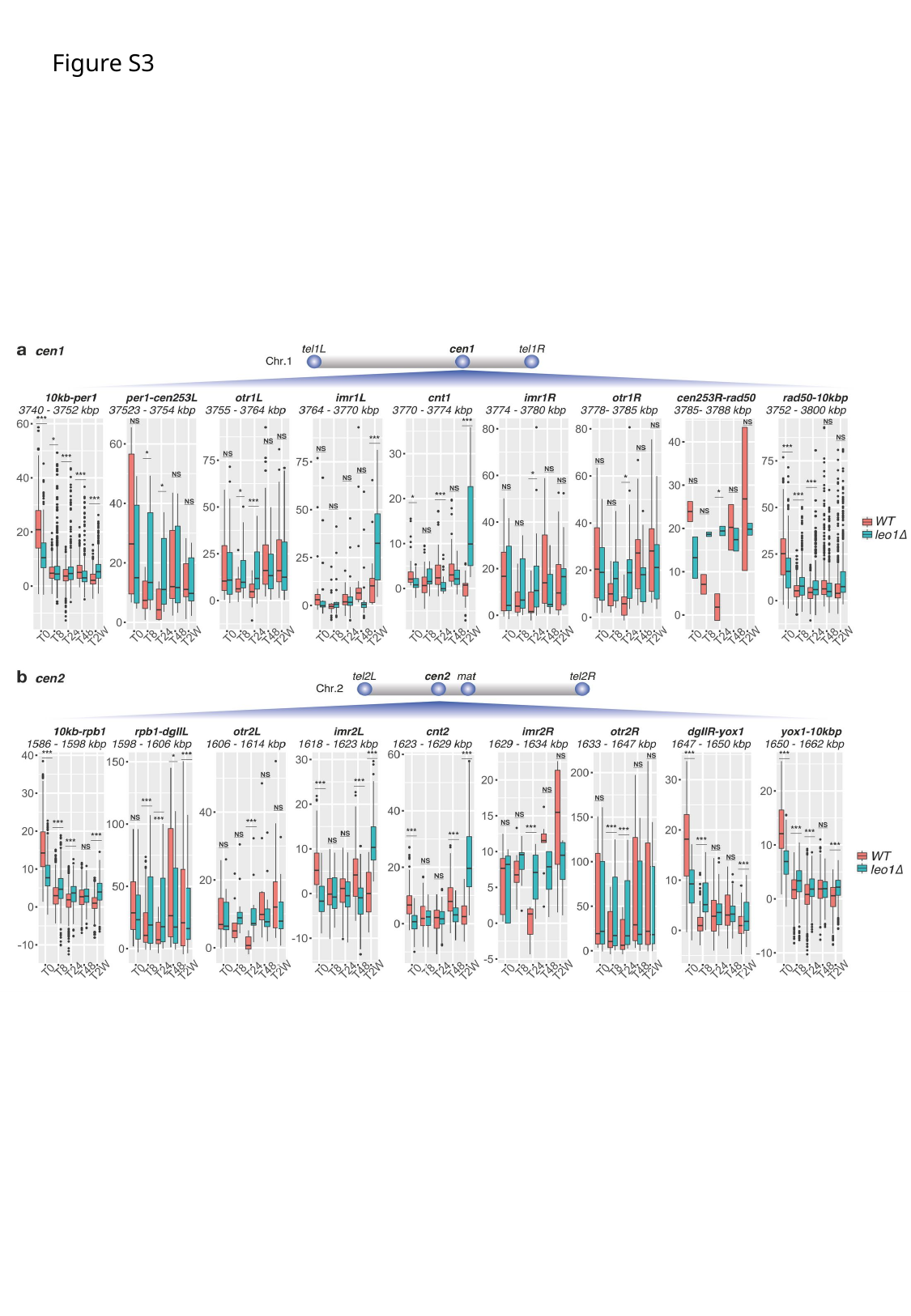

Figure S3

## Slide 3
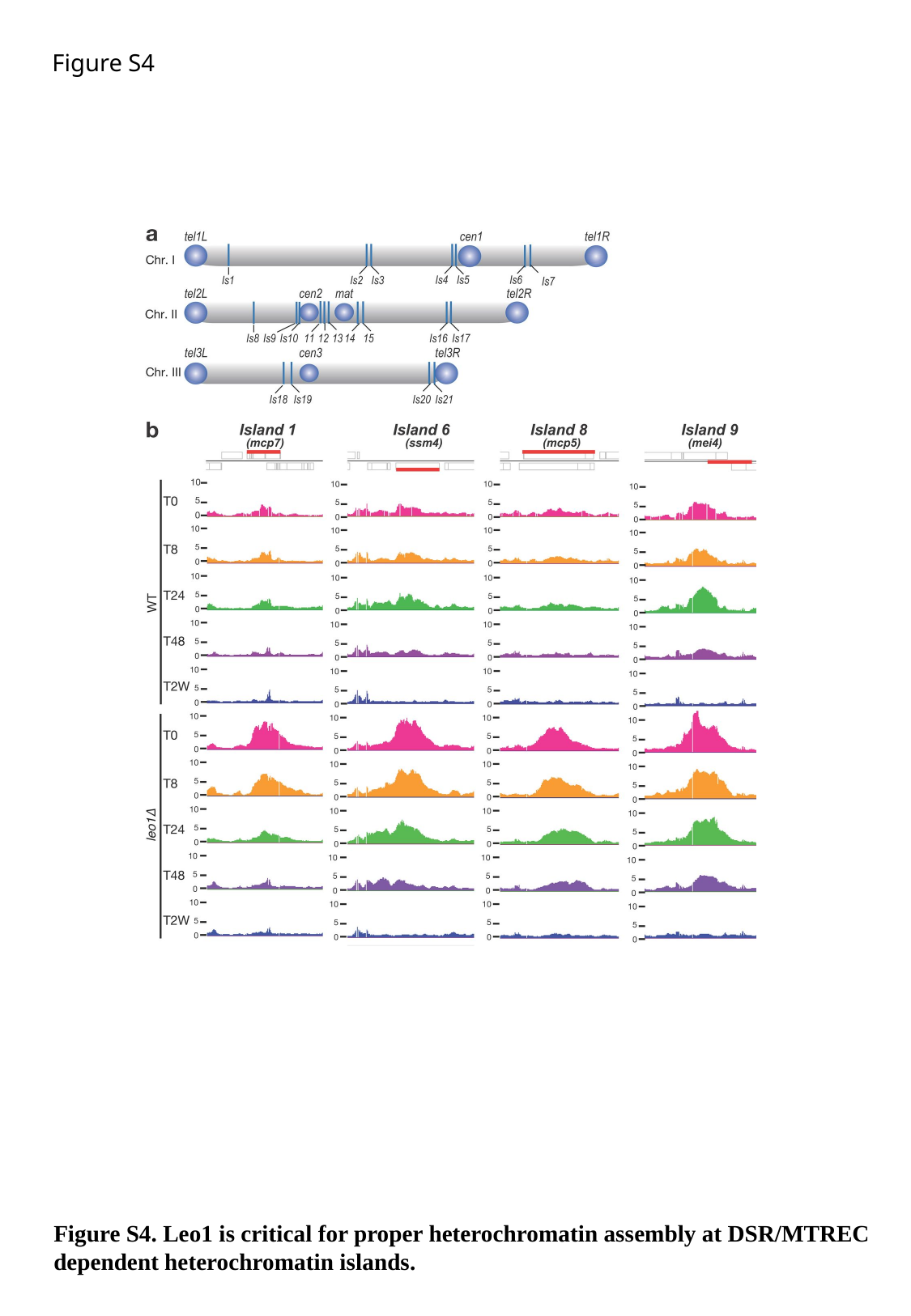

Figure S4
Figure S4. Leo1 is critical for proper heterochromatin assembly at DSR/MTREC
dependent heterochromatin islands.

## Slide 4
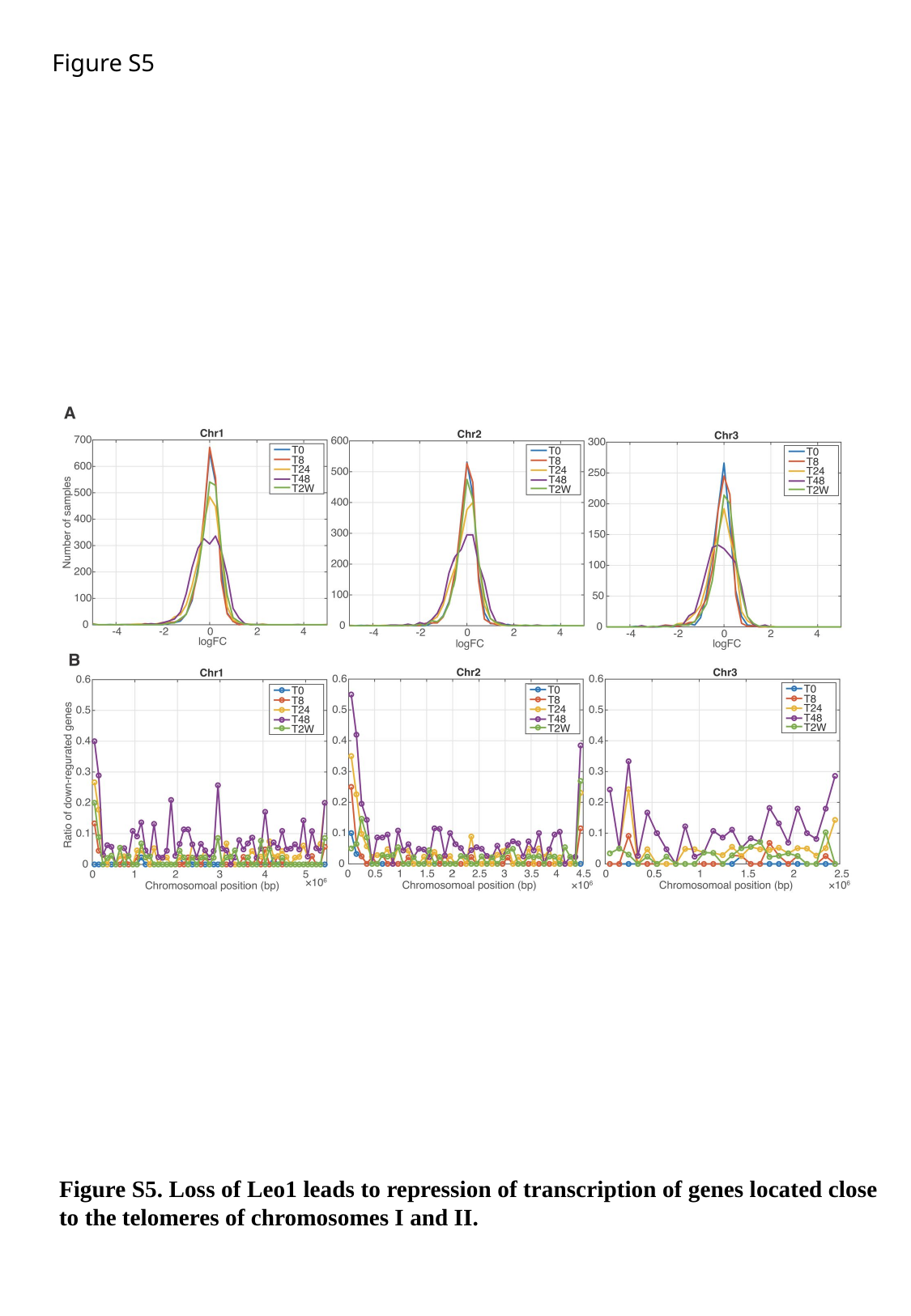

Figure S5
Figure S5. Loss of Leo1 leads to repression of transcription of genes located close to the telomeres of chromosomes I and II.

## Slide 5
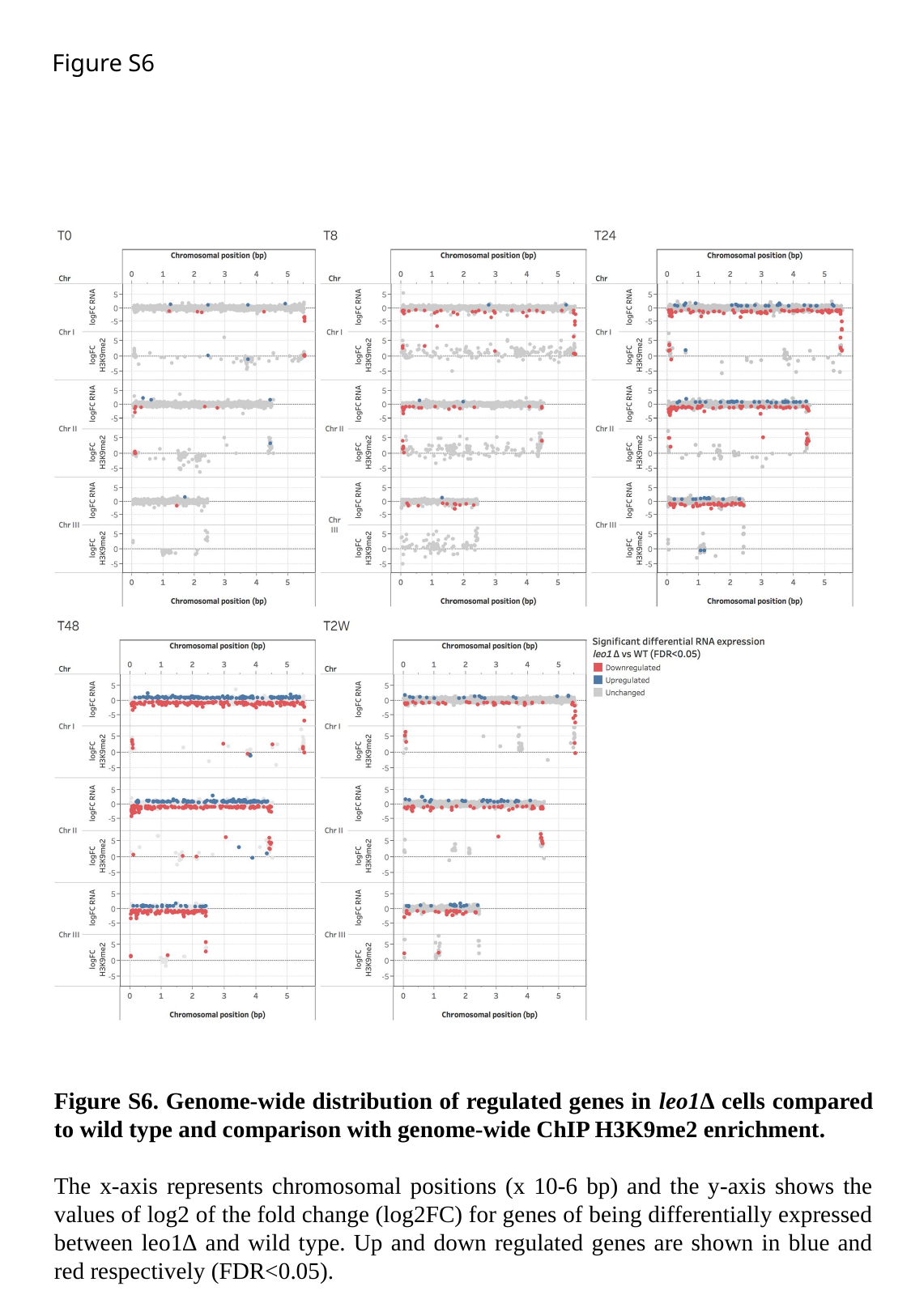

Figure S6
Figure S6. Genome-wide distribution of regulated genes in leo1∆ cells compared to wild type and comparison with genome-wide ChIP H3K9me2 enrichment.
The x-axis represents chromosomal positions (x 10-6 bp) and the y-axis shows the values of log2 of the fold change (log2FC) for genes of being differentially expressed between leo1∆ and wild type. Up and down regulated genes are shown in blue and red respectively (FDR<0.05).

## Slide 6
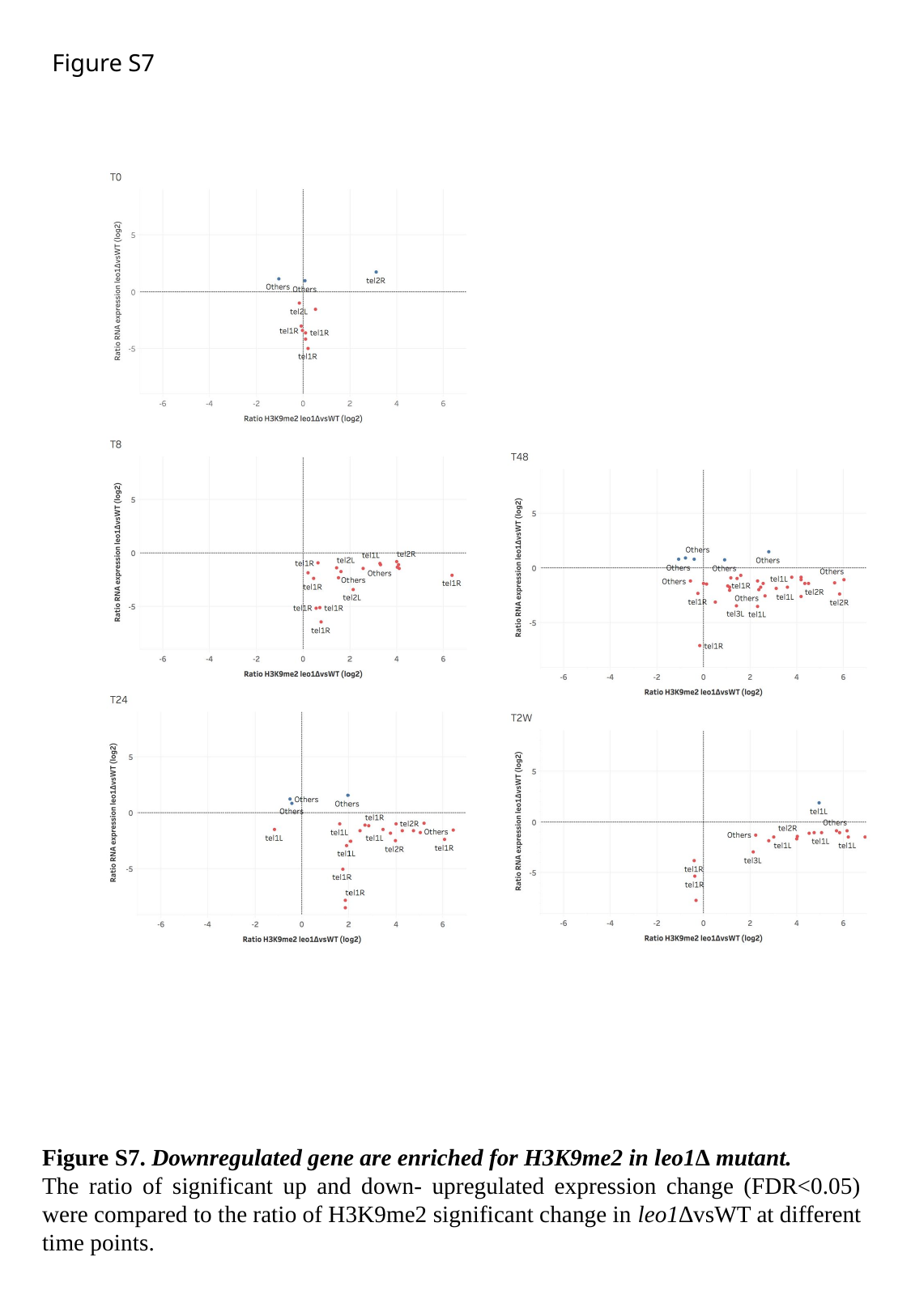

Figure S7
Figure S7. Downregulated gene are enriched for H3K9me2 in leo1∆ mutant.
The ratio of significant up and down- upregulated expression change (FDR<0.05) were compared to the ratio of H3K9me2 significant change in leo1∆vsWT at different time points.

## Slide 7
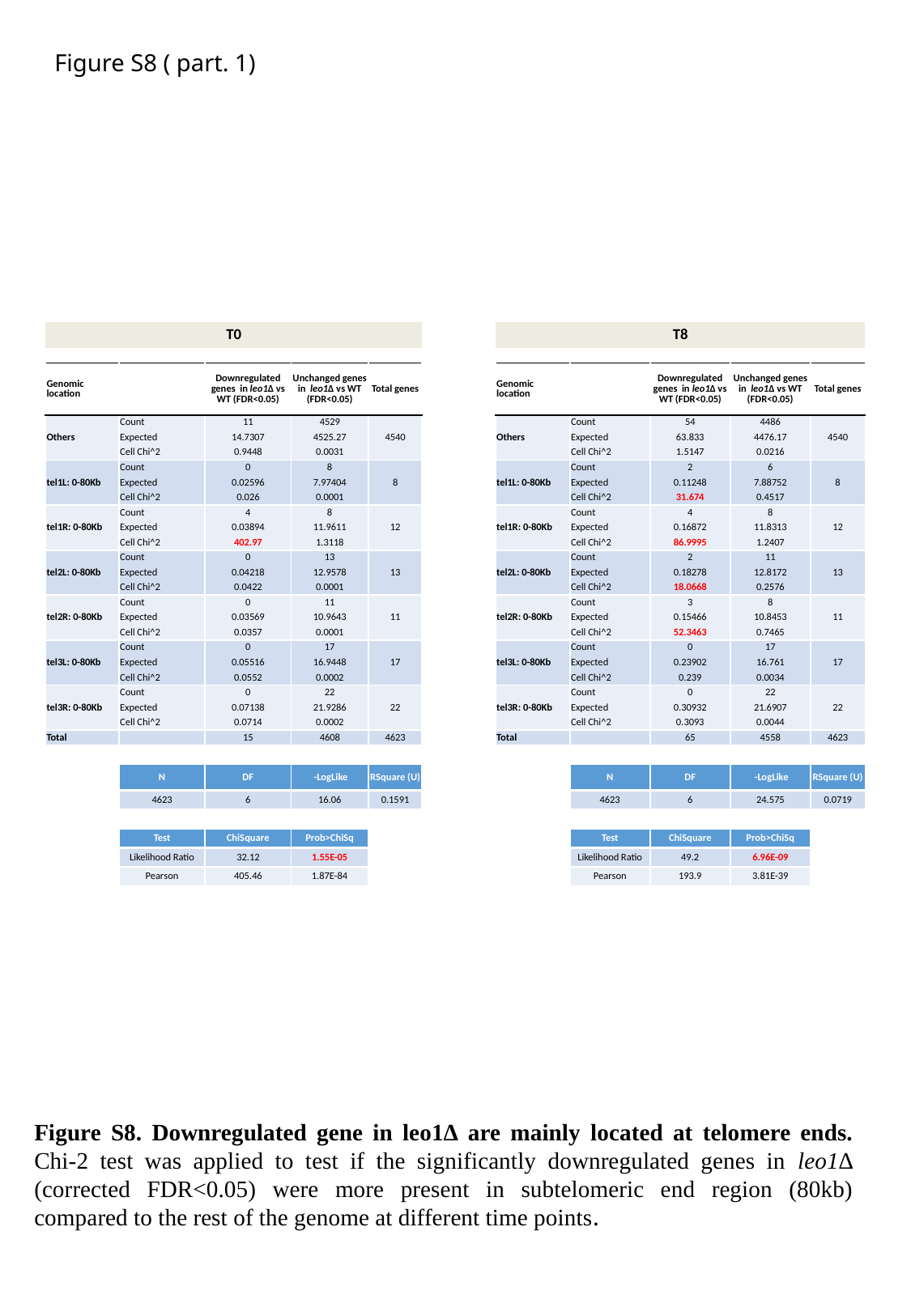

Figure S8 ( part. 1)
| | | | | | | | | | | |
| --- | --- | --- | --- | --- | --- | --- | --- | --- | --- | --- |
| | | | | | | | | | | |
| T0 | | | | | | T8 | | | | |
| | | | | | | | | | | |
| Genomic location | | Downregulated genes in leo1∆ vs WT (FDR<0.05) | Unchanged genes in leo1∆ vs WT (FDR<0.05) | Total genes | | Genomic location | | Downregulated genes in leo1∆ vs WT (FDR<0.05) | Unchanged genes in leo1∆ vs WT (FDR<0.05) | Total genes |
| Others | Count | 11 | 4529 | 4540 | | Others | Count | 54 | 4486 | 4540 |
| | Expected | 14.7307 | 4525.27 | | | | Expected | 63.833 | 4476.17 | |
| | Cell Chi^2 | 0.9448 | 0.0031 | | | | Cell Chi^2 | 1.5147 | 0.0216 | |
| tel1L: 0-80Kb | Count | 0 | 8 | 8 | | tel1L: 0-80Kb | Count | 2 | 6 | 8 |
| | Expected | 0.02596 | 7.97404 | | | | Expected | 0.11248 | 7.88752 | |
| | Cell Chi^2 | 0.026 | 0.0001 | | | | Cell Chi^2 | 31.674 | 0.4517 | |
| tel1R: 0-80Kb | Count | 4 | 8 | 12 | | tel1R: 0-80Kb | Count | 4 | 8 | 12 |
| | Expected | 0.03894 | 11.9611 | | | | Expected | 0.16872 | 11.8313 | |
| | Cell Chi^2 | 402.97 | 1.3118 | | | | Cell Chi^2 | 86.9995 | 1.2407 | |
| tel2L: 0-80Kb | Count | 0 | 13 | 13 | | tel2L: 0-80Kb | Count | 2 | 11 | 13 |
| | Expected | 0.04218 | 12.9578 | | | | Expected | 0.18278 | 12.8172 | |
| | Cell Chi^2 | 0.0422 | 0.0001 | | | | Cell Chi^2 | 18.0668 | 0.2576 | |
| tel2R: 0-80Kb | Count | 0 | 11 | 11 | | tel2R: 0-80Kb | Count | 3 | 8 | 11 |
| | Expected | 0.03569 | 10.9643 | | | | Expected | 0.15466 | 10.8453 | |
| | Cell Chi^2 | 0.0357 | 0.0001 | | | | Cell Chi^2 | 52.3463 | 0.7465 | |
| tel3L: 0-80Kb | Count | 0 | 17 | 17 | | tel3L: 0-80Kb | Count | 0 | 17 | 17 |
| | Expected | 0.05516 | 16.9448 | | | | Expected | 0.23902 | 16.761 | |
| | Cell Chi^2 | 0.0552 | 0.0002 | | | | Cell Chi^2 | 0.239 | 0.0034 | |
| tel3R: 0-80Kb | Count | 0 | 22 | 22 | | tel3R: 0-80Kb | Count | 0 | 22 | 22 |
| | Expected | 0.07138 | 21.9286 | | | | Expected | 0.30932 | 21.6907 | |
| | Cell Chi^2 | 0.0714 | 0.0002 | | | | Cell Chi^2 | 0.3093 | 0.0044 | |
| Total | | 15 | 4608 | 4623 | | Total | | 65 | 4558 | 4623 |
| | | | | | | | | | | |
| | N | DF | -LogLike | RSquare (U) | | | N | DF | -LogLike | RSquare (U) |
| | 4623 | 6 | 16.06 | 0.1591 | | | 4623 | 6 | 24.575 | 0.0719 |
| | | | | | | | | | | |
| | Test | ChiSquare | Prob>ChiSq | | | | Test | ChiSquare | Prob>ChiSq | |
| | Likelihood Ratio | 32.12 | 1.55E-05 | | | | Likelihood Ratio | 49.2 | 6.96E-09 | |
| | Pearson | 405.46 | 1.87E-84 | | | | Pearson | 193.9 | 3.81E-39 | |
Figure S8. Downregulated gene in leo1∆ are mainly located at telomere ends. Chi-2 test was applied to test if the significantly downregulated genes in leo1∆ (corrected FDR<0.05) were more present in subtelomeric end region (80kb) compared to the rest of the genome at different time points.

## Slide 8
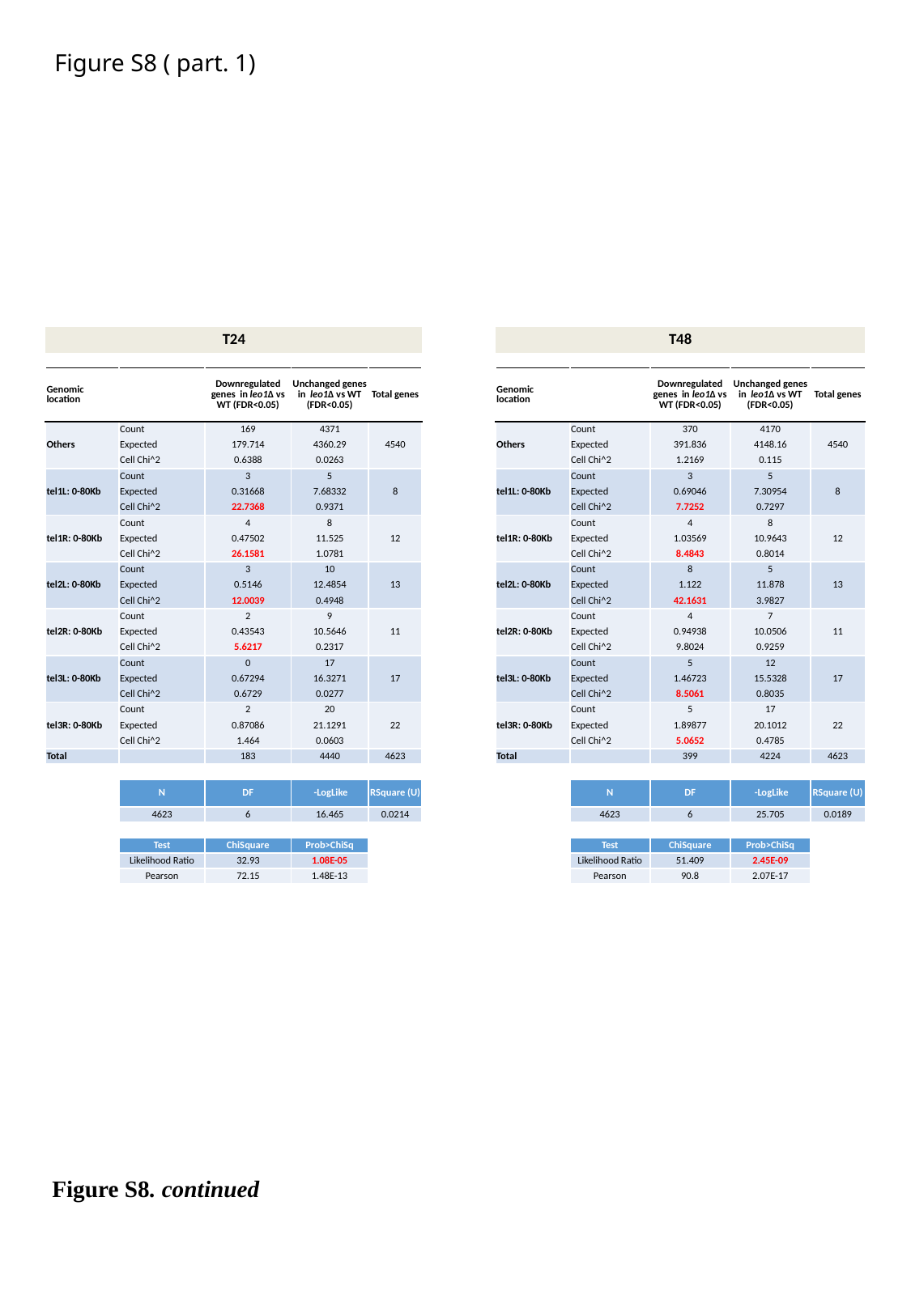

Figure S8 ( part. 1)
| T24 | | | | | | T48 | | | | |
| --- | --- | --- | --- | --- | --- | --- | --- | --- | --- | --- |
| | | | | | | | | | | |
| Genomic location | | Downregulated genes in leo1∆ vs WT (FDR<0.05) | Unchanged genes in leo1∆ vs WT (FDR<0.05) | Total genes | | Genomic location | | Downregulated genes in leo1∆ vs WT (FDR<0.05) | Unchanged genes in leo1∆ vs WT (FDR<0.05) | Total genes |
| Others | Count | 169 | 4371 | 4540 | | Others | Count | 370 | 4170 | 4540 |
| | Expected | 179.714 | 4360.29 | | | | Expected | 391.836 | 4148.16 | |
| | Cell Chi^2 | 0.6388 | 0.0263 | | | | Cell Chi^2 | 1.2169 | 0.115 | |
| tel1L: 0-80Kb | Count | 3 | 5 | 8 | | tel1L: 0-80Kb | Count | 3 | 5 | 8 |
| | Expected | 0.31668 | 7.68332 | | | | Expected | 0.69046 | 7.30954 | |
| | Cell Chi^2 | 22.7368 | 0.9371 | | | | Cell Chi^2 | 7.7252 | 0.7297 | |
| tel1R: 0-80Kb | Count | 4 | 8 | 12 | | tel1R: 0-80Kb | Count | 4 | 8 | 12 |
| | Expected | 0.47502 | 11.525 | | | | Expected | 1.03569 | 10.9643 | |
| | Cell Chi^2 | 26.1581 | 1.0781 | | | | Cell Chi^2 | 8.4843 | 0.8014 | |
| tel2L: 0-80Kb | Count | 3 | 10 | 13 | | tel2L: 0-80Kb | Count | 8 | 5 | 13 |
| | Expected | 0.5146 | 12.4854 | | | | Expected | 1.122 | 11.878 | |
| | Cell Chi^2 | 12.0039 | 0.4948 | | | | Cell Chi^2 | 42.1631 | 3.9827 | |
| tel2R: 0-80Kb | Count | 2 | 9 | 11 | | tel2R: 0-80Kb | Count | 4 | 7 | 11 |
| | Expected | 0.43543 | 10.5646 | | | | Expected | 0.94938 | 10.0506 | |
| | Cell Chi^2 | 5.6217 | 0.2317 | | | | Cell Chi^2 | 9.8024 | 0.9259 | |
| tel3L: 0-80Kb | Count | 0 | 17 | 17 | | tel3L: 0-80Kb | Count | 5 | 12 | 17 |
| | Expected | 0.67294 | 16.3271 | | | | Expected | 1.46723 | 15.5328 | |
| | Cell Chi^2 | 0.6729 | 0.0277 | | | | Cell Chi^2 | 8.5061 | 0.8035 | |
| tel3R: 0-80Kb | Count | 2 | 20 | 22 | | tel3R: 0-80Kb | Count | 5 | 17 | 22 |
| | Expected | 0.87086 | 21.1291 | | | | Expected | 1.89877 | 20.1012 | |
| | Cell Chi^2 | 1.464 | 0.0603 | | | | Cell Chi^2 | 5.0652 | 0.4785 | |
| Total | | 183 | 4440 | 4623 | | Total | | 399 | 4224 | 4623 |
| | | | | | | | | | | |
| | N | DF | -LogLike | RSquare (U) | | | N | DF | -LogLike | RSquare (U) |
| | 4623 | 6 | 16.465 | 0.0214 | | | 4623 | 6 | 25.705 | 0.0189 |
| | | | | | | | | | | |
| | Test | ChiSquare | Prob>ChiSq | | | | Test | ChiSquare | Prob>ChiSq | |
| | Likelihood Ratio | 32.93 | 1.08E-05 | | | | Likelihood Ratio | 51.409 | 2.45E-09 | |
| | Pearson | 72.15 | 1.48E-13 | | | | Pearson | 90.8 | 2.07E-17 | |
Figure S8. continued

## Slide 9
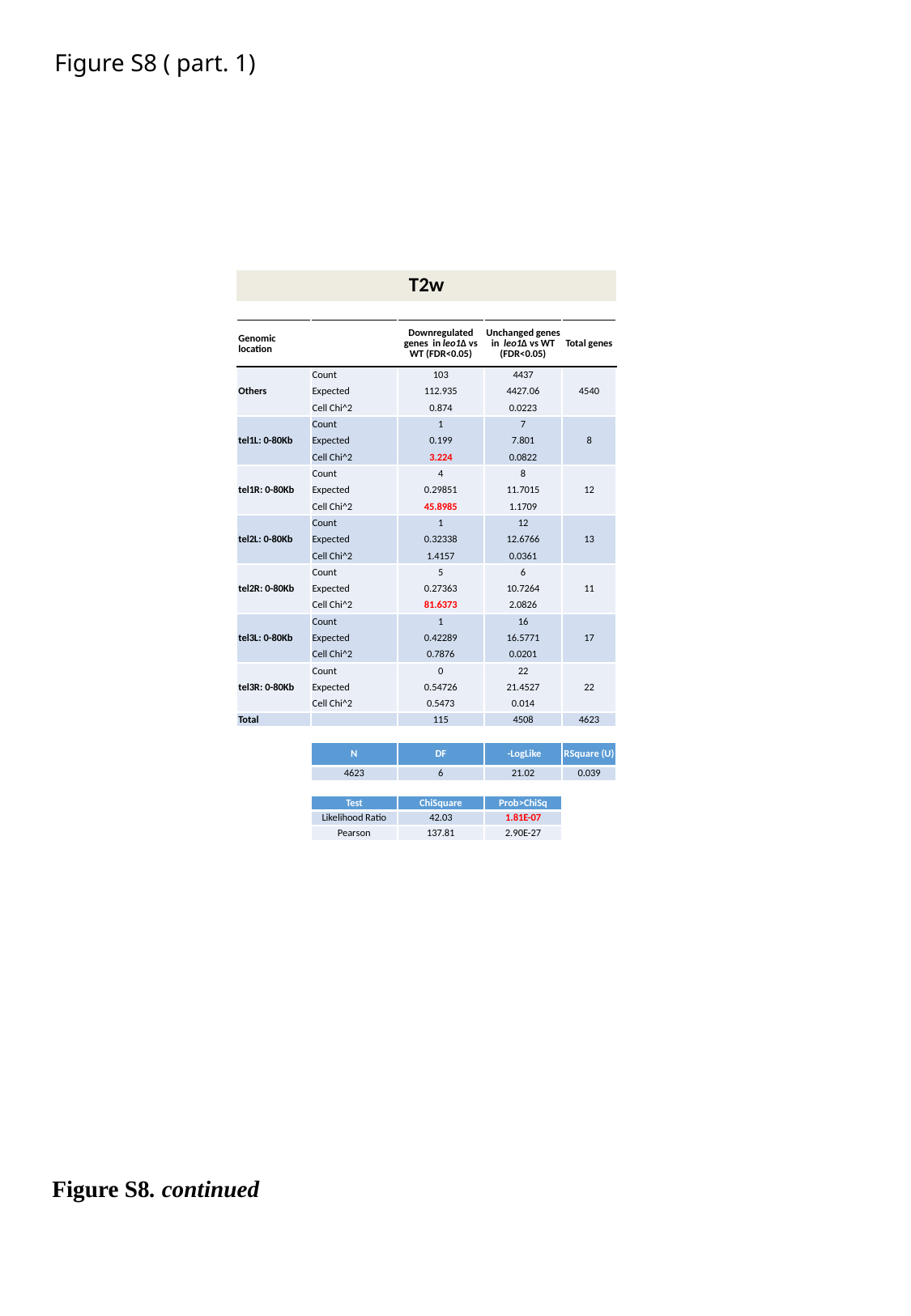

Figure S8 ( part. 1)
| T2w | | | | |
| --- | --- | --- | --- | --- |
| | | | | |
| Genomic location | | Downregulated genes in leo1∆ vs WT (FDR<0.05) | Unchanged genes in leo1∆ vs WT (FDR<0.05) | Total genes |
| Others | Count | 103 | 4437 | 4540 |
| | Expected | 112.935 | 4427.06 | |
| | Cell Chi^2 | 0.874 | 0.0223 | |
| tel1L: 0-80Kb | Count | 1 | 7 | 8 |
| | Expected | 0.199 | 7.801 | |
| | Cell Chi^2 | 3.224 | 0.0822 | |
| tel1R: 0-80Kb | Count | 4 | 8 | 12 |
| | Expected | 0.29851 | 11.7015 | |
| | Cell Chi^2 | 45.8985 | 1.1709 | |
| tel2L: 0-80Kb | Count | 1 | 12 | 13 |
| | Expected | 0.32338 | 12.6766 | |
| | Cell Chi^2 | 1.4157 | 0.0361 | |
| tel2R: 0-80Kb | Count | 5 | 6 | 11 |
| | Expected | 0.27363 | 10.7264 | |
| | Cell Chi^2 | 81.6373 | 2.0826 | |
| tel3L: 0-80Kb | Count | 1 | 16 | 17 |
| | Expected | 0.42289 | 16.5771 | |
| | Cell Chi^2 | 0.7876 | 0.0201 | |
| tel3R: 0-80Kb | Count | 0 | 22 | 22 |
| | Expected | 0.54726 | 21.4527 | |
| | Cell Chi^2 | 0.5473 | 0.014 | |
| Total | | 115 | 4508 | 4623 |
| | | | | |
| | N | DF | -LogLike | RSquare (U) |
| | 4623 | 6 | 21.02 | 0.039 |
| | | | | |
| | Test | ChiSquare | Prob>ChiSq | |
| | Likelihood Ratio | 42.03 | 1.81E-07 | |
| | Pearson | 137.81 | 2.90E-27 | |
Figure S8. continued

## Slide 10
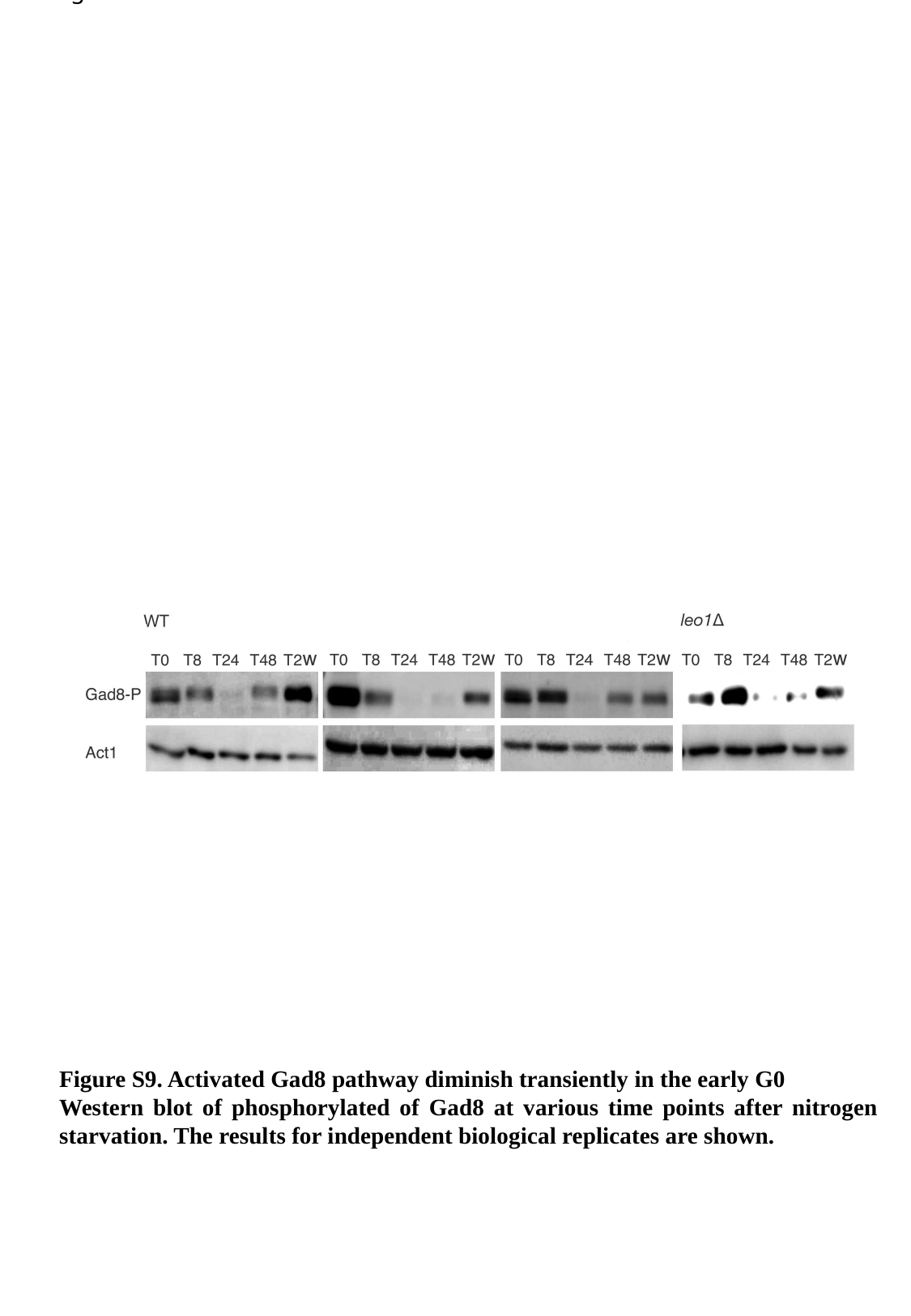

Figure S9
Figure S9. Activated Gad8 pathway diminish transiently in the early G0
Western blot of phosphorylated of Gad8 at various time points after nitrogen starvation. The results for independent biological replicates are shown.

## Slide 11
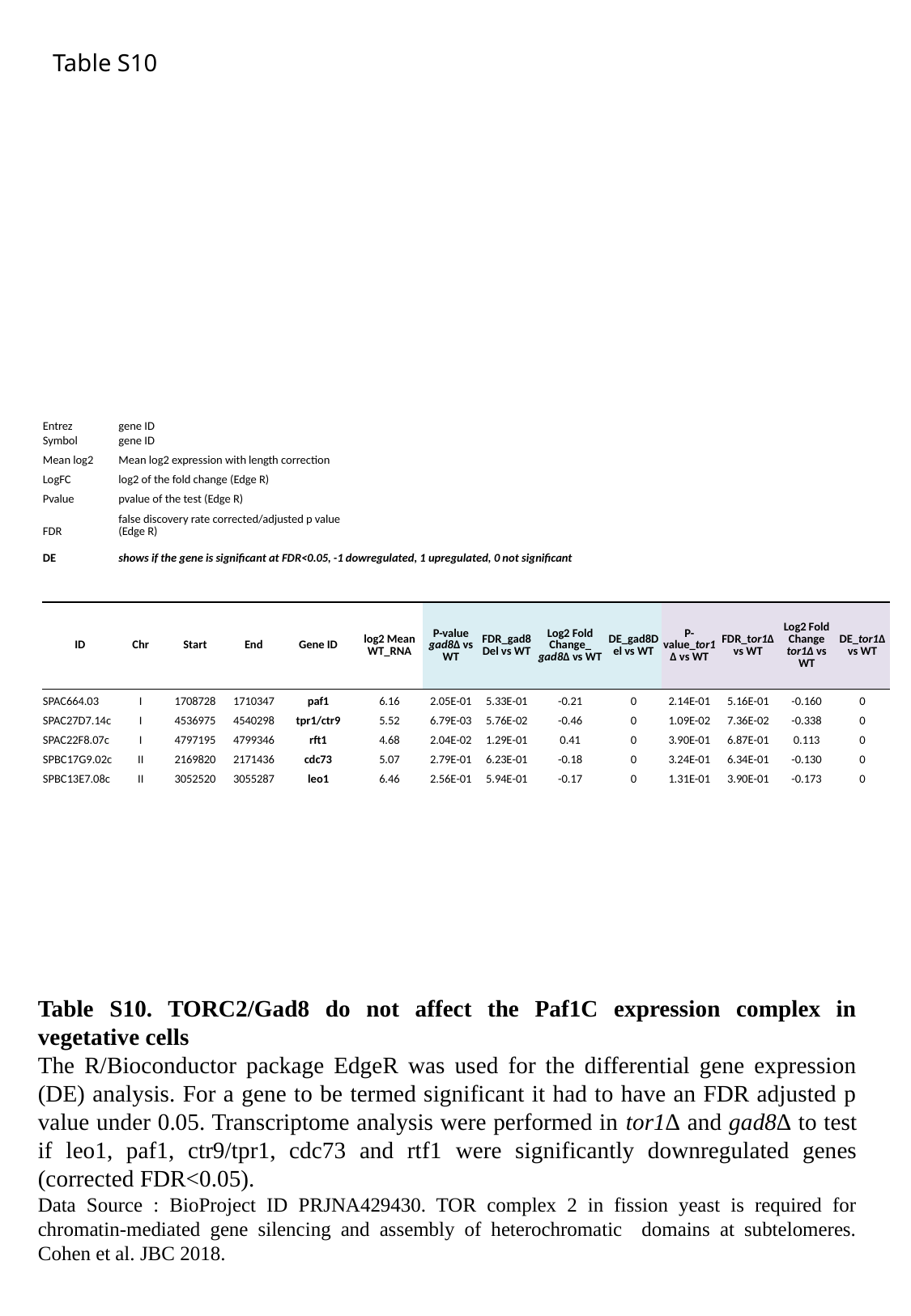

Table S10
| Entrez | gene ID | | | | | | | | | | | | | | |
| --- | --- | --- | --- | --- | --- | --- | --- | --- | --- | --- | --- | --- | --- | --- | --- |
| Symbol | gene ID | | | | | | | | | | | | | | |
| Mean log2 | Mean log2 expression with length correction | | | | | | | | | | | | | | |
| LogFC | log2 of the fold change (Edge R) | | | | | | | | | | | | | | |
| Pvalue | pvalue of the test (Edge R) | | | | | | | | | | | | | | |
| FDR | false discovery rate corrected/adjusted p value (Edge R) | | | | | | | | | | | | | | |
| DE | shows if the gene is significant at FDR<0.05, -1 dowregulated, 1 upregulated, 0 not significant | | | | | | | | | | | | | | |
| | | | | | | | | | | | | | | | |
| ID | Chr | Start | | End | Gene ID | log2 Mean WT\_RNA | | P-value gad8∆ vs WT | FDR\_gad8Del vs WT | Log2 Fold Change\_ gad8∆ vs WT | DE\_gad8Del vs WT | P-value\_tor1∆ vs WT | FDR\_tor1∆ vs WT | Log2 Fold Change tor1∆ vs WT | DE\_tor1∆ vs WT |
| SPAC664.03 | I | 1708728 | | 1710347 | paf1 | 6.16 | | 2.05E-01 | 5.33E-01 | -0.21 | 0 | 2.14E-01 | 5.16E-01 | -0.160 | 0 |
| SPAC27D7.14c | I | 4536975 | | 4540298 | tpr1/ctr9 | 5.52 | | 6.79E-03 | 5.76E-02 | -0.46 | 0 | 1.09E-02 | 7.36E-02 | -0.338 | 0 |
| SPAC22F8.07c | I | 4797195 | | 4799346 | rft1 | 4.68 | | 2.04E-02 | 1.29E-01 | 0.41 | 0 | 3.90E-01 | 6.87E-01 | 0.113 | 0 |
| SPBC17G9.02c | II | 2169820 | | 2171436 | cdc73 | 5.07 | | 2.79E-01 | 6.23E-01 | -0.18 | 0 | 3.24E-01 | 6.34E-01 | -0.130 | 0 |
| SPBC13E7.08c | II | 3052520 | | 3055287 | leo1 | 6.46 | | 2.56E-01 | 5.94E-01 | -0.17 | 0 | 1.31E-01 | 3.90E-01 | -0.173 | 0 |
Table S10. TORC2/Gad8 do not affect the Paf1C expression complex in vegetative cells
The R/Bioconductor package EdgeR was used for the differential gene expression (DE) analysis. For a gene to be termed significant it had to have an FDR adjusted p value under 0.05. Transcriptome analysis were performed in tor1∆ and gad8∆ to test if leo1, paf1, ctr9/tpr1, cdc73 and rtf1 were significantly downregulated genes (corrected FDR<0.05).
Data Source : BioProject ID PRJNA429430. TOR complex 2 in fission yeast is required for chromatin-mediated gene silencing and assembly of heterochromatic domains at subtelomeres. Cohen et al. JBC 2018.
