# Supplementary material for: Leo1 is essential for the dynamic regulation of heterochromatin and gene expression during cellular quiescence
Source: Epigenetics Chromatin. 2019 Jul 17;12:45. doi: 10.1186/s13072-019-0292-7 (PMC6636030; doi:10.1186/s13072-019-0292-7)
Supplement: Supplementary file 3 — Additional file 3. Supplementary data Figure S10. [file 13072_2019_292_MOESM3_ESM.pptx]

## Slide 1
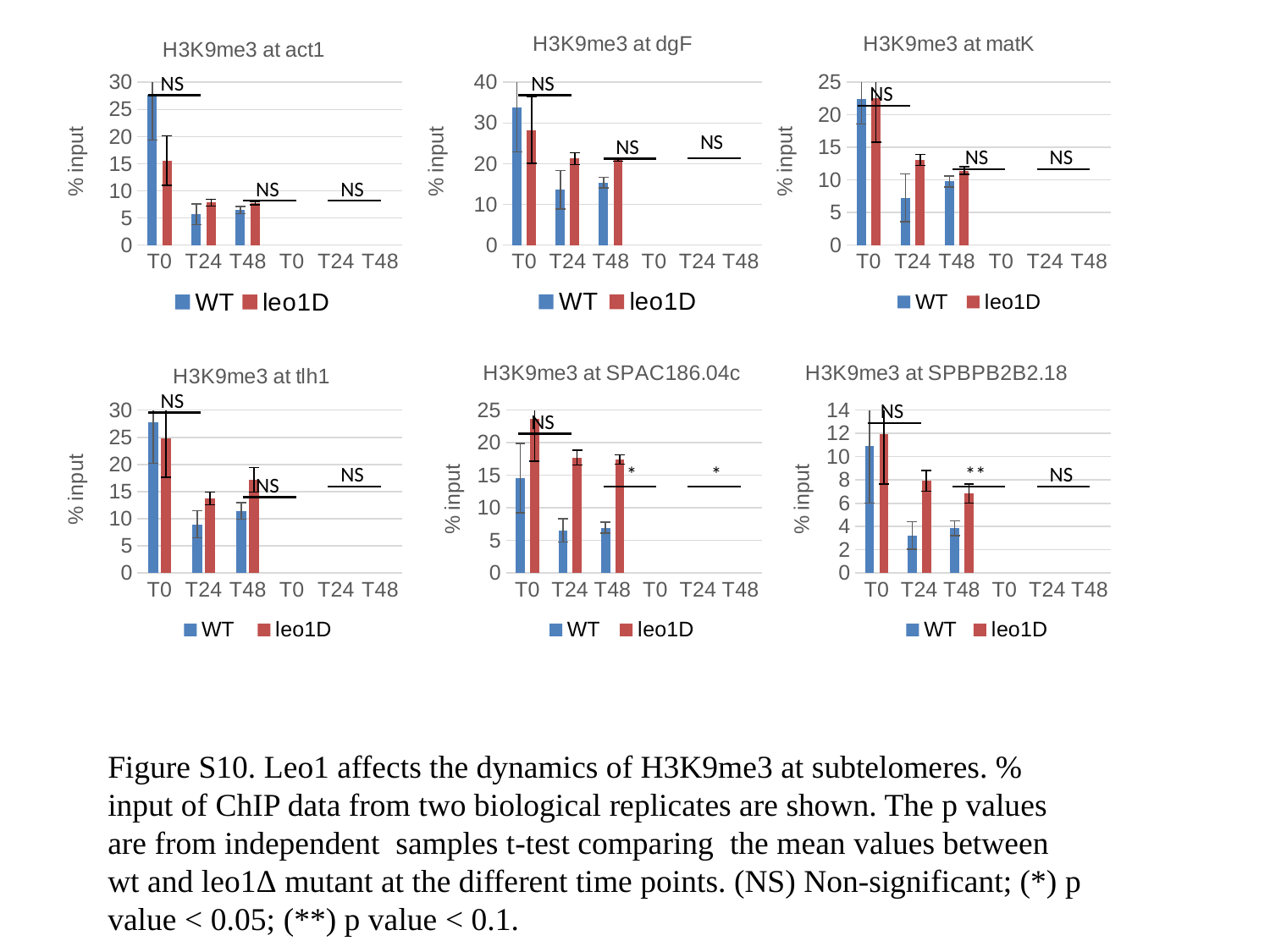

[unsupported chart]
[unsupported chart]
[unsupported chart]
NS
NS
NS
NS
NS
NS
NS
NS
[unsupported chart]
[unsupported chart]
[unsupported chart]
NS
NS
NS
NS
*
*
**
NS
NS
Figure S10. Leo1 affects the dynamics of H3K9me3 at subtelomeres. % input of ChIP data from two biological replicates are shown. The p values are from independent samples t-test comparing the mean values between wt and leo1Δ mutant at the different time points. (NS) Non-significant; (*) p value < 0.05; (**) p value < 0.1.
